# Identification of rare cortical folding patterns using unsupervised deep learning

**DOI:** 10.1162/imag_a_00084

**Published:** 2024-02-06

**Authors:** Louise Guillon, Joël Chavas, Audrey Bénézit, Marie-Laure Moutard, Pauline Roca, Charles Mellerio, Catherine Oppenheim, Denis Rivière, Jean-François Mangin

**Affiliations:** CEA, CNRS, NeuroSpin, Baobab, Université Paris-Saclay, Gif-sur-Yvette, France; Service de Neurologie et Réanimation Pédiatrique, Hôpital Raymond Poincaré, APHP, Garches, France; Service de Neuropédiatrie, Hôpital Trousseau, Hôpitaux Universitaires de l’Est Parisien, Sorbonne Université, Paris, France; Institut de Psychiatrie et Neurosciences de Paris (IPNP), INSERM, UMR S1266, Université de Paris, Paris, France; Imaging Department, Groupe Hospitalier Universitaire Paris Psychiatrie et Neurosciences, Sainte-Anne Hospital, Paris, France; Pixyl, Research and Development Laboratory, Grenoble, France; Centre d’imagerie du Nord, Saint Denis, France

**Keywords:** Folding patterns, cortical folding, cortical sulci, anomaly detection, unsupervised learning, β − VAE, epilepsy

## Abstract

Like fingerprints, cortical folding patterns are unique to each brain even though they follow a general species-specific organization. Some folding patterns have been linked with neurodevelopmental disorders. However, due to the high inter-individual variability, the identification of rare folding patterns that could become biomarkers remains a very complex task. This paper proposes a novel unsupervised deep learning approach to identify rare folding patterns and assess the degree of deviations that can be detected. To this end, we preprocess the brain MR images to focus the learning on the folding morphology and train a beta variational auto-encoder (β−VAE) on the inter-individual variability of the folding to identify outliers. We compare the detection power of the latent space and of the reconstruction errors, using synthetic benchmarks and one actual rare configuration related to the central sulcus. Finally, we assess the generalization of our method on a developmental anomaly located in another region and we validate the relevance of our approach on patients suffering from drug-resistant epilepsy. Our results suggest that this method enables encoding relevant folding characteristics that can be enlightened and better interpreted based on the generative power of the β−VAE. The latent space and the reconstruction errors bring complementary information and enable the identification of rare patterns of different nature. This method generalizes well to a different region on another dataset and demonstrates promising results on the epileptic patients. Code is available at https://github.com/neurospin-projects/2022_lguillon_rare_folding_detection.

## 1 Introduction

**Fig. 1.**
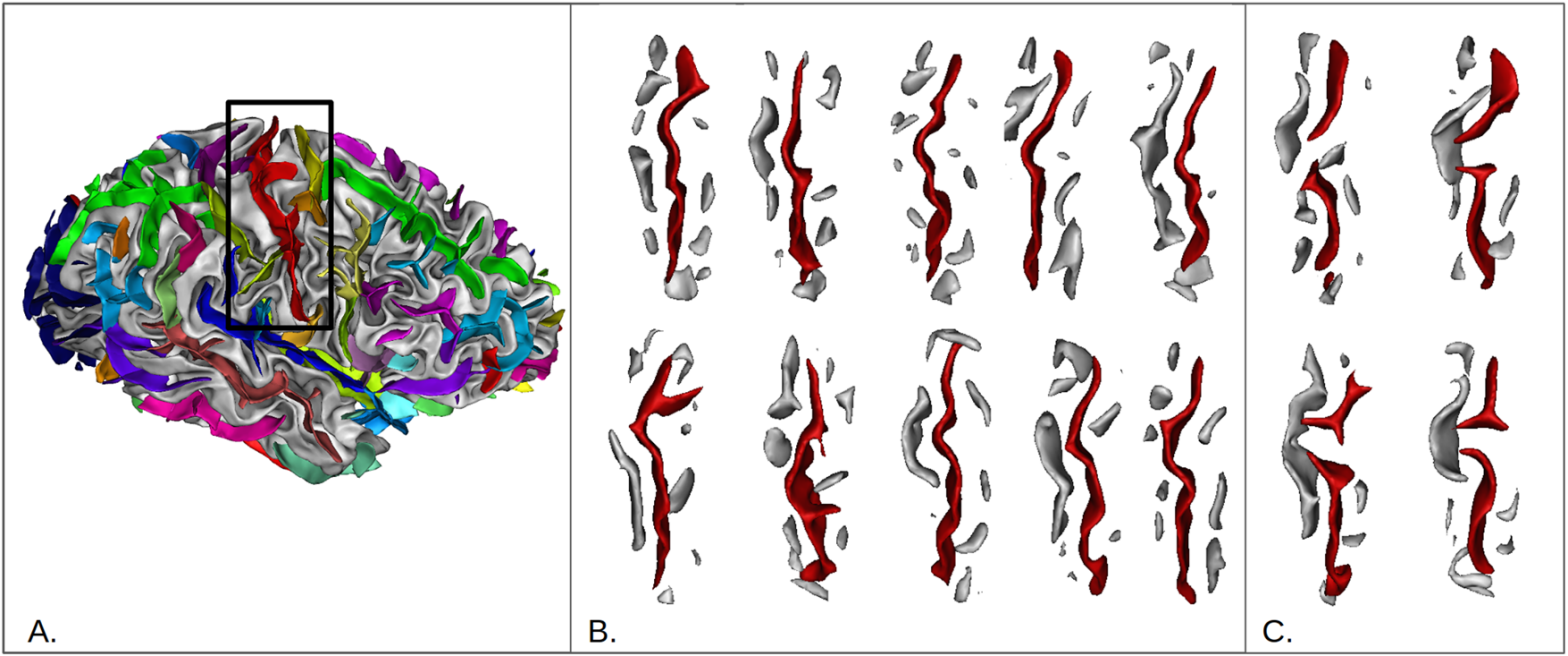
*Central sulcus region variability.* (A) Localization of the studied region of interest (ROI) on a 3D view of one right hemisphere. The colored ribbons represent sulci, defined as a negative cast of the furrows. The central sulcus is red. (B) Examples of non-interrupted central sulci. (C) Examples of interrupted central sulci.

During gestation, the human cortex folds and gets its convoluted shape composed of gyri—the ridges of white matter—that are delimited by furrows—the sulci. In the human population, stability of the folding patterns is observed with an overall similarity of location, shape, and arrangements (Ono et al., 1990). This stability is important enough to define a nomenclature of sulci and to develop methods that automate sulci recognition (Borne et al., 2020; Rivière et al., 2002). Despite this homogeneity, each brain displays a unique cortical folding, acting as a fingerprint (Wachinger et al., 2015). Figure 1A and B show examples of the variability in the central sulcus region, which is one of the most stable.

The folding variability is so complex that it has long been overlooked. However, thanks to advances in the neuroimaging field, studies have tried to characterize sulci with elementary shapes that amount to building blocks of alternative patterns. In that sense, a knob or a flat segment are shapes that can be combined to form patterns, such as single knob, double knob, or flat patterns. For instance, the central sulcus is typically composed of one or several knobs (Yousry et al., 1997). In contrast, some very rare patterns have also been described, like the interruption of the central sulcus that can be found in only about 1% of the population (Mangin et al., 2019) (see Fig. 1C).

Folding patterns have proved to be very interesting as they are related to function. For example, the central sulcus divides the cortex into the motor and the sensory areas, and specific parts of the central sulcus have been correlated with the cortical areas of the tongue, foot, and hand among others (Germann et al., 2020; Mangin et al., 2019). In particular, the central sulcus main knob has been linked to the hand motricity and is called the “hand knob” (Yousry et al., 1997).

Specific patterns were also correlated to neurodevelopmental disorders. The Power Button Sign (PBS), a rare configuration of the precentral sulcus, may be associated with a certain type of epilepsy (Mellerio et al., 2014). Patterns in the superior temporal sulcus, central, intraparietal, and frontal regions could be related to autism (Auzias et al., 2014; Hotier et al., 2017; Levitt et al., 2003). Hence, deciphering sulcal complexity and having a better understanding of the underlying shape variability is of great interest: folding patterns could become biomarkers of neurodevelopmental disorders.

Advances in machine learning and deep learning, in particular, are now opening up new possibilities for studying folding patterns, identifying typical or rare patterns, and hopefully, emerging sulcal biomarkers. Indeed, we pointed out the tremendous inter-individual variability which makes the identification of deviating patterns highly complex. Thanks to its representation power, deep learning can prove to be a very effective way to model inter-individual variability in order to spot rare folding configurations. In particular, unsupervised deep learning constitutes a real opportunity for this task for several reasons. First, only a few rare patterns have been described, making supervised algorithms not suited for the task: training the algorithms on a very few number of cases is challenging and we seek to identify *unknown* rare patterns. Moreover, the identification of some of the rare patterns, such as the PBS, was done visually on a small cohort. However, doing it visually is highly difficult and patterns specific to patients of a given cohort may be present in controls of other cohorts. This may be suggested by the results of the automatic detection of PBS in an all control population, where a CNN-based model identified a PBS for 30% of the hemispheres (Borne et al., 2021). Although some of these hemispheres were wrongly classified, it highlights that (1) the PBS is also present in a healthy population and that (2) its identification is not a trivial problem. As a consequence, using an unsupervised strategy on a wider dataset constitutes an opportunity to address these problems and identify rare folding patterns.

In this work, we investigate whether an unsupervised deep learning model can identify deviating regional patterns; and if so, what granularity of deviations can be detected? Here, we define granularity as the characteristics and properties of the anomalies, such as their size or nature. The analysis of the granularity aims to characterize the abnormal features that can be detected and at what level of detail. We also seek to describe which space is the most relevant to identify deviating patterns: is it based on the reconstruction error, in the input space, that is to say in our case, the *folding space*, or is it the latent space?

Folding mechanisms may lead to both global and regional anomalies and these two scales have led to correlations with function disorders (Fernández et al., 2016). Here, we focus on regional patterns rather than on a global representation. Specifically, we concentrate on the central sulcus which is a good candidate for our work. Indeed, it is one of the first folds to appear and it is stable enough to be a first step in modeling inter-individual variability. More importantly, usually long and continuous, the central sulcus can be interrupted in very rare cases, making interrupted central sulci relevant patterns to assess our method. Finally, this region is of clinical interest as it is linked to hand motricity and asymmetries have been described (Bo et al., 2015; Sun et al., 2012).

To perform our study, we worked on the HCP database (Van Essen et al., 2013). From the MR images, we focused on the folding morphology of the central sulcus area with a specific preprocessing that does not require labeling the sulci of the studied subjects. Indeed, in the future, we wish to be able to apply our methodology to new databases whether the sulci are labeled or not. We then trained a β−VAE to learn the inter-individual variability. Due to the small number of interrupted central sulci and to be able to characterize the detected granularity, we designed synthetic benchmarks of rare patterns to assess our methodology more reliably. We then investigated the detection power of our methodology both on the latent space and on the *folding space*, using either our synthetic outliers or interrupted central sulci, an actual rare pattern. The synthetic benchmarks serve as a proof of concept indicating the feasibility of the approach and providing information on the granularity of what can be detected. To assess the generalization, we applied our approach on another dataset showing an abnormal folding pattern in a different brain region, the cingulate sulcus. Finally, we validated our approach on patients with drug-resistant epilepsy, whom we attempted to distiguish from controls and identify specific sulcal features.

## 2 Literature Review

Folding patterns can be analyzed with two main approaches. On one hand, morphometric features can be extracted such as the depth, the surface curvature, or the opening of each sulcus. On the other hand, one can look directly at the shapes of the sulci. Working on shapes rather than on morphometric values is particularly interesting as they constitute “trait features” opposite to “state features” (Cachia et al., 2016). Unlike state features that can evolve during the lifespan, trait features remain fixed after birth. For example, the sulcal opening is a state feature because it increases with aging (Jin et al., 2018; Kochunov et al., 2005). In return, the pattern of the cingulate sulcus area is a trait feature because it is stable throughout life after infancy. Different strategies can be adopted for exploring the shapes: a finite number of shapes can be considered, using clustering for instance (Duan et al., 2019; Meng et al., 2018), or shapes can be represented in a continuous way, such as manifold-based analyses (Sun et al., 2012; de Vareilles et al., 2022). For both strategies, a first step is required to represent the folding patterns. Folding shapes’ complexity can be reduced based on the similarity between different sulci (Sun et al., 2009, 2012; de Vareilles et al., 2022) based on the Wasserstein distance after registration of the sulci for instance, or between sulcal graphs (Im et al., 2011; Meng et al., 2018) based on multiple metrics characterizing the folding metrics. These similarity measures are then either directly analyzed, or projected to a lower dimensional space.

Recently, deep learning approaches have also been proposed to study folding patterns. In particular, two unsupervised deep learning models, a β−VAE and SimCLR, were compared in the task of identifying typical patterns in the cingulate region (Guillon et al., 2022). Other works have focused on the task of identifying abnormal folding patterns thanks to unsupervised deep learning in the region of the superior temporal sulcus branches (Guillon et al., 2021).

Anomaly and outlier detection has been a subject of great interest in the domain of biomedical imaging: many studies, including for brain MR images, have tried to identify abnormal samples (Chalapathy & Chawla, 2019; Fernando et al., 2022). A common framework is to use auto-encoders as they implement a latent space with fewer dimensions than the input which makes it hard to encode uncommon features. Then, the identification of anomalies is usually performed based on the reconstruction error rather than in the latent space.

## 3 Materials and Methods

### 3.1 Database

We used T1-weighted MR images of the Human Connectome Project (HCP) dataset (Van Essen et al., 2013). Data were acquired on a single Siemens Skyra Connectom scanner at an isotropic resolution of 0.7 mm. Subjects are healthy controls from 22 to 36 years old.

The long-term goal of this work is to identify rare folding patterns that have not been characterized yet. However, we first need to assess our method. To do so, we worked on a rare pattern already described, the interrupted central sulci (CS). A previous study identified in this database seven sulci in the right hemisphere and two in the left (Auzias et al., 2015; Mangin et al., 2019). We chose to work on the right hemisphere rather than on the left in order to have the highest number of rare patterns and we kept only right-handed subjects leading to a total of 1001 subjects.

### 3.2 Folding representation

**Fig. 2.**
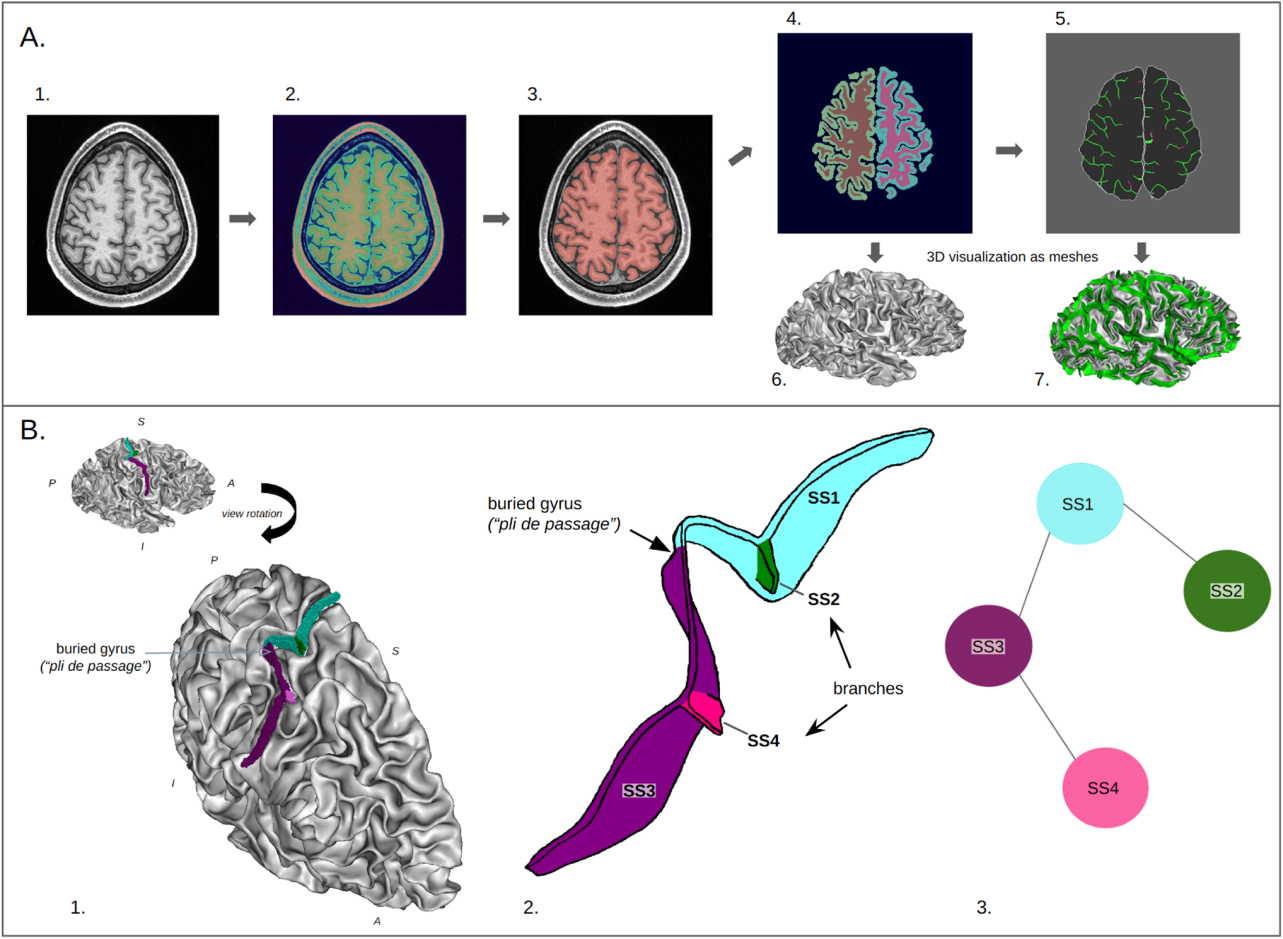
*Overview of the BrainVISA/Morphologist pipeline’s main steps and of the folds representation.* (A) *Main steps of BrainVISA/Morphologist pipeline.* 1. Raw T1-w MRI, 2. Bias-corrected image, 3. Segmentation of the brain, 4. Segmentation of the hemispheres and of the grey and white matter, 5. Skeleton representation of the folding graph, representing a negative cast of the 4. 6. Mesh representation of the white matter of the right hemisphere, 7. Folding graph that represents the folds (in green) as the negative cast of the white matter of the right hemisphere (white mesh).(B) *Folds representation.* 1. Example of a central sulcus, which is composed of several elementary entities called simple surfaces (SS). (Orientation: A: Anterior, P: Posterior, S: Superior, I: Inferior). 2. Corresponding schematic representation of the sulcus represented in 1, which is formed by four simple surfaces. Depth variation caused by the buried gyrus and the presence of two branches lead to the division into four different simple surfaces. 3. Corresponding folding graph.

In this work, we consider the folds as the skeleton of a negative cast of the brain, that is to say, voxels located in the cerebrospinal fluid, (Fig. 2A.5) (Mangin et al., 1995), which can be represented as ribbons located between the gyri (green ribbons in Fig. 2A.7). Folding or sulcal patterns are defined as the combination and arrangements of shapes of one or several folds.

**Fig. 3.**
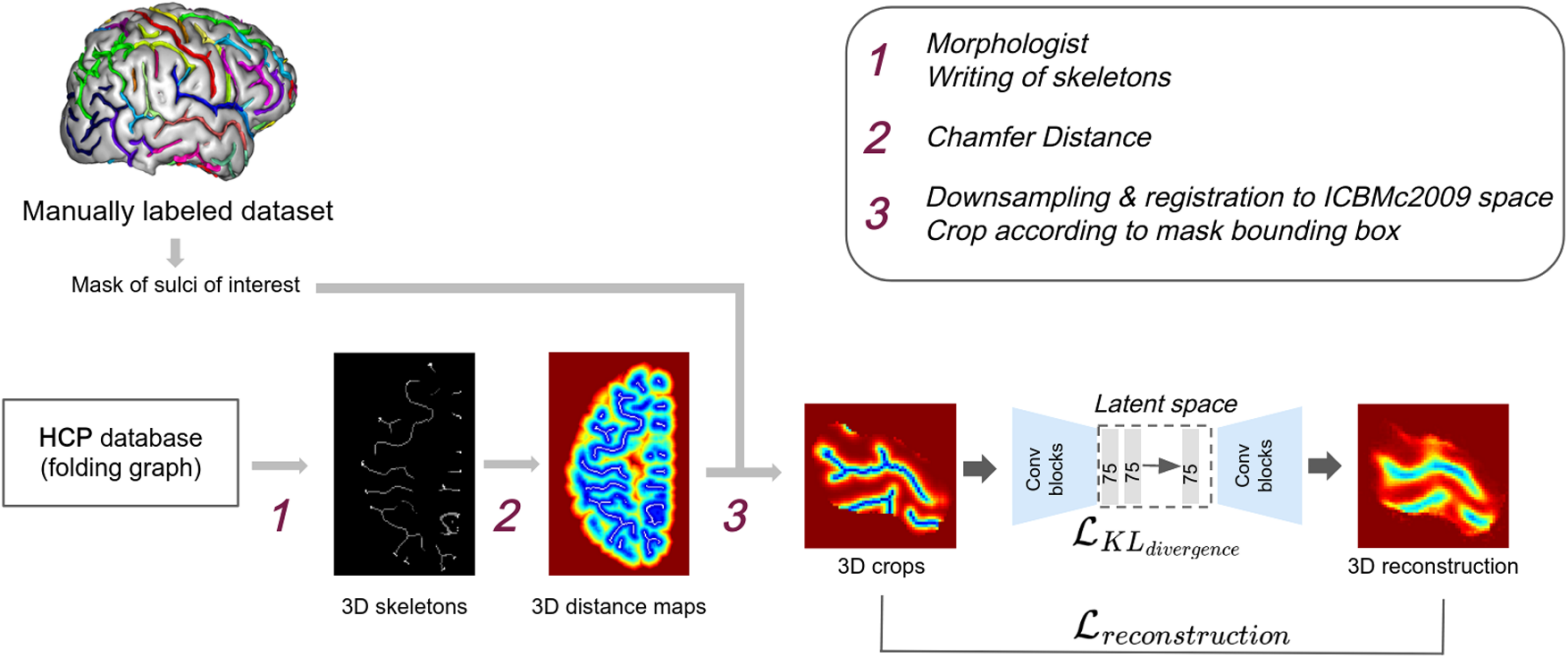
*Pipeline.* A mask of the central sulcus area is defined based on a distinct manually labeled dataset. HCP is processed with Morphologist to obtain folding graphs, which are used to obtain 3D images of skeletons (1). The Chamfer distance is applied to the skeletons to obtain geodesic distance maps (2). Distance maps are then downsampled, registered to the ICBMc2009 space, cropped according to the mask (3), and fed as input to a β−VAE. Labeled sulci are only used to define the masks once, before any preprocessing and training. Once the masks are defined on the external dataset, no labels are used to model inter-individual variability and identify outliers.

*BrainVisa/Morphologist pipeline.* Structural MR images hold numerous pieces of information beyond the morphology of cortical folding. In order to focus on the folding characteristics, we developed a preprocessing pipeline. The raw MR images are first processed by the BrainVISA/Morphologist software (https://brainvisa.info/). This pipeline is composed of several steps that include skull stripping, bias correction, segmentation of the brain and of the hemispheres, skeletonization of the grey matter, and the cerebrospinal fluid union (Fig. 2A) (Rivière et al., 2002). This step leads to so-called skeletons, 3D images representing only the folding which is then segmented into simple surfaces (SS) depending on various parameters such as the sulcal depth or topological properties (Fig. 2B) (Mangin et al., 1995). All in all, the obtained outputs are 3D images that correspond to a negative cast of the brain (first step of Fig. 3).

*From skeletons to distance maps*. Skeleton-based images have proved to be relevant and were the object of previous studies for fold recognition (Borne et al., 2020, 2021), as well as for folding patterns representation (Guillon et al., 2021, 2022). However, in this work, we applied an additional preprocessing step to convert our skeleton images into distance maps as we believe that skeletons have several shortcomings that could limit the performance of our approach. First, skeletons are binary images representing the folding patterns; hence, they are very sparse images and only very few voxels hold explicit sulcal information in skeletons. On average, sulci represent less than 5% of the voxels in our region of interest (ROI). We argue that it would be interesting to have a representation where the information devoted to folding patterns is more distributed. In addition, skeleton images are not smooth and the local regions of interest in our application correspond to high frequencies making the skeleton details harder to represent and reconstruct. It is also complex to reconstruct folding patterns in skeletons as they are not continuous images, so there is no notion of the proximity of a voxel to a sulcus. This makes the reconstruction error and the gradient-based learning less efficient. Finally, distance maps are built based on the whole hemisphere; therefore, if we work on an ROI, they give, especially near the border of the ROI, information about objects outside the ROI. Therefore, to tackle these shortcomings, we convert the resulting skeletons into distance maps based on the Chamfer distance, which approximates the Euclidean distance. Sulci are considered objects, and the further away a sulcus is, the larger the value of a voxel is. An example of a distance map is presented in Figure 3 step 2. To visualize the folds, distance maps are converted to meshes (see Annex 3 of the Supplementary Information).

*Focusing on a single region: crop definition.* As we are interested in capturing the local folding patterns variability, such as the hand knob, rather than the global hemisphere-wide arrangements of sulci, we chose to focus our study on a sub-region of the right hemisphere, the central sulcus area. To define the ROI, we learned a mask of the central sulcus over a manually labeled dataset comprising 62 healthy controls (Borne et al., 2020). Subjects are first affinely registered to the ICBMc2009 space and resampled to an isotropic resolution of 1 mm. Then, for each subject, a mask in the ICMBc2009 space is incremented for all the voxels of the central sulcus represented as a set of simple surfaces. The resulting mask is slightly dilated by 5 mm to include potential central sulcus locations not represented in our database. HCP subjects’ distance maps are affinely registered to the ICBMc2009 space and then cropped according to the mask bounding box (step 3 of Fig. 3). The mask is applied on the fly during the training of our network. It is important to note that the sulci labels are only used for obtaining the mask coordinates but are not needed afterwards, once the masks are defined. Therefore, when applying our approach to the HCP subjects or to any other dataset, sulci labels are neither required nor used.

### 3.3 Learning a representation of the folding variability

#### 3.3.1 beta-VAE

In order to identify rare patterns we first seek to model the inter-individual variability. In the outlier detection field based on unsupervised methods, auto-encoder (AE) models are widely used as they implement a latent space, also known as the bottleneck, that has far fewer dimensions than the input space. Usually, training is performed only on control subjects. The assumption is that the model learns a representation of the *normal* variability and that at inference when facing outliers, it will not be able to encode and reconstruct them as well as control data. To overcome some shortcomings of simple convolutional AE and to regularize the latent space, variational auto-encoder (VAE) was introduced (Kingma & Welling, 2014). Its strength also lies in its generative power, enabling not only to reconstruct but also to generate new data, which is particularly interesting for interpretability purposes. Other AE-based models have been used for anomaly detection in the biomedical field such as Generative Adversarial Networks (GAN) (Schlegl et al., 2017, 2019), which were then transposed to brain images (Simarro Viana et al., 2021). A comparison of AE models showed that VAE was one of the most efficient in brain MR images (Baur et al., 2020). In the context of representation learning of folding patterns, VAE was proved to be well adapted (Guillon et al., 2021, 2022).

In the VAE framework, a sample of input space X is mapped to a distribution in a latent space Z of L dimensions, by an encoder θ. A vector z is then drawn from this distribution and reconstructed by a decoder ϕ. The objective function seeks to minimize both the reconstruction error and the Kullback-Leibler (KL) divergence ($D_{KL}$). The model is thus trained to maximize:

$$ℒ\left(\theta ,\phi ;x,z,\beta \right)=E_{q_{\phi }(z|x)}[\text{log}p_{\theta }(x|z)]-\beta D_{KL}(q_{\phi }(z|x)||p(z))$$ (1)

where p(z) refers to the prior distribution (in this work, a reduced centered Gaussian distribution) which is approximated with $q_{\phi }(z|x)$, the posterior distribution. β−VAE is an extension of the VAE where the KL divergence is weighted by β (Higgins et al., 2017).

#### 3.3.2 Training procedure

##### 3.3.2.1 Preprocessing

The input data of the model are the previously defined just cropped, then masked distance maps. For augmentation purposes, random rotations between [-10°, 10°], centered on the mask center, are drawn from a uniform distribution at each epoch and applied to the whole brain, before applying the mask that strictly remains at the same position. Such rotations are also sought to limit the edge effects. More precisely, the central sulcus is surrounded by two main folds, the precentral and the postcentral sulci. Parts of these sulci are included in the ROI. Therefore, rotating the distance map *under* the mask enables to capture a wider context and to try to limit their influence. We apply a normalization on the distance maps to have values between [0, 1], with the highest values on the folds and a saturation at about 4-5 mm which corresponds to half of the typical width of gyri. Details are presented in Annex 1 of the Supplementary Information. Finally, we apply a small padding, resulting in samples of dimensions 80 x 80 x 96.

##### 3.3.2.2 Training

Dataset was split into train, validation, and test sets of respectively 640, 161, and 200 subjects. Training is only performed on control data, that is, subjects with continuous central sulci, all identified interrupted central sulci (CS) were added to the test set. However, we point out that there may remain some undetected interrupted central sulci in the training set as all subjects were not individually inspected. To model the normal inter-individual variability, we used a classic convolutional β−VAE of depth 3. In order to choose the best values for β and latent space dimension L, we performed a gridsearch (β= 2-10, L = 4-150). Each parameter configuration was assessed based on two criteria. Our first criterion is the reconstruction quality. Indeed, we seek to leverage the reconstruction and generative power of the β−VAE, hence the reconstruction quality must be sufficient. Our second criterion is the detection power on a proxy for the interrupted central sulci. The pre-central and post-central sulci demonstrate some similarities with the central sulcus in terms of orientation, size, and shape. However, they tend to be more interrupted and to present a higher number of ramifications. Therefore, we used the HCP dataset crops of these two other regions as fake outliers for our gridsearch. We selected only pre- and post-central sulci which presented some ambiguities with the central sulcus based on the procedure described in Annex 4 of the Supplementary Information. For each hyperparameter combination, we trained a β−VAE on the train set and classified between the latent codes of the validation samples and of the pre- or post-central sulci. We kept the hyperparameters that led to the best classification results and good reconstructions (based on reconstruction error and visual inspection). Details are presented in Annex 4 of the Supplementary Information. The number of training epochs is determined based on early stopping.

### 3.4 Generating synthetic rare patterns

One of the challenges of our work is the lack of consensual rare patterns to evaluate our methodology. In addition, it would be interesting to be able to quantify the degree of deviation that our model is able to detect. Therefore, several sets of synthetic rare patterns were generated to be used as benchmarks. Both benchmarks were generated from the test set subjects.

#### 3.4.1 Deletion benchmark

Our first benchmark consists of subjects for whom we have erased one simple surface (SS). Erasing small simple surfaces could be a good proxy to simulate rare patterns because some fold branches may be missing in some people, or a sulcus may be shorter or absent. Large simple surfaces are less likely to be missing but allow us to assess the degree of deviation that can be detected.

**Fig. 4.**
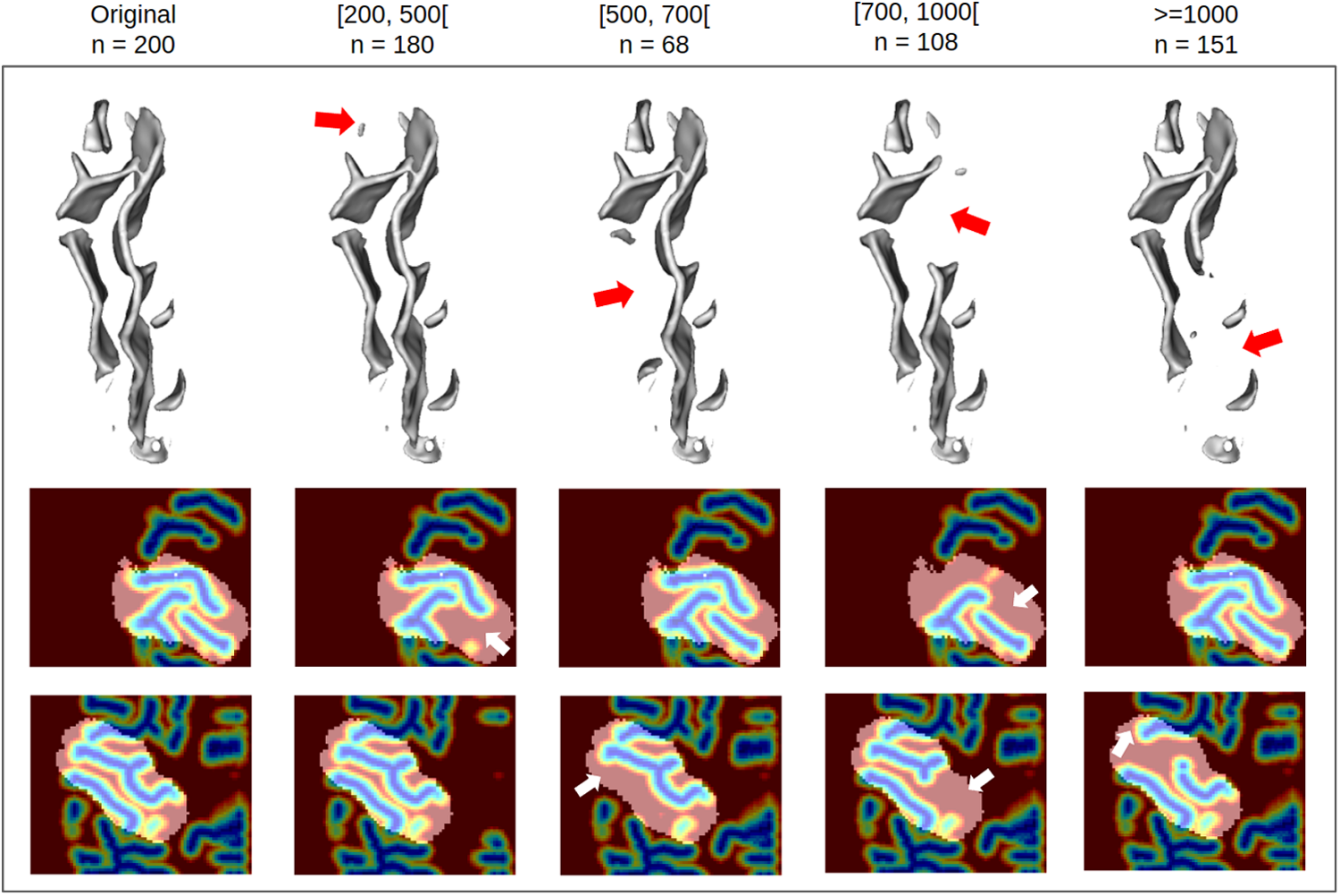
*Deletion benchmarks.* Visualization of original sulcal pattern and its altered versions from the four deletion benchmarks showing patterns with increasing simple surface size deleted. Upper row: Mesh visualization. Middle and bottom rows: distance maps on axial view, visualization at depths 15 and 37.

To analyze the granularity of anomaly that can be detected by our method, we generated several benchmarks which vary according to the size of the deleted simple surface (SS). As such, we created four sets where SS size was between 200-500 voxels, 500-700 voxels, 700-1000 voxels, and simple surfaces of more than 1000 voxels. In the following, we name each set with the minimum number of voxels: for instance, *200* corresponds to the benchmark where simple surfaces of size between 200 and 500 were erased. To be deleted, simple surfaces must have a number of voxels included inside the mask corresponding to the range of the different sets. If several simple surfaces meet the criteria, one is randomly chosen to be erased. Otherwise, a subject may not have a simple surface satisfying the requirements. In such cases, the subject is not included in the benchmarks. Finally, from the 200 test subjects, benchmark 200 contains 180 subjects; benchmark 500, 68; benchmark 700, 108; and benchmark 1000, 151 subjects. The smaller simple surfaces are mostly part of the precentral and postcentral sulci, representing more than 85% of the surfaces between 200 and 500 voxels. On the contrary, larger simple surfaces correspond to the central sulcus (see Annex 2 of the Supplementary Information). Therefore, beyond deleting simple surfaces of varying sizes, the nature of the sulci, and thus the location are also different, especially between the set 200 and the others. An example is presented in Figure 4.

#### 3.4.2 Asymmetry benchmark

**Fig. 5.**
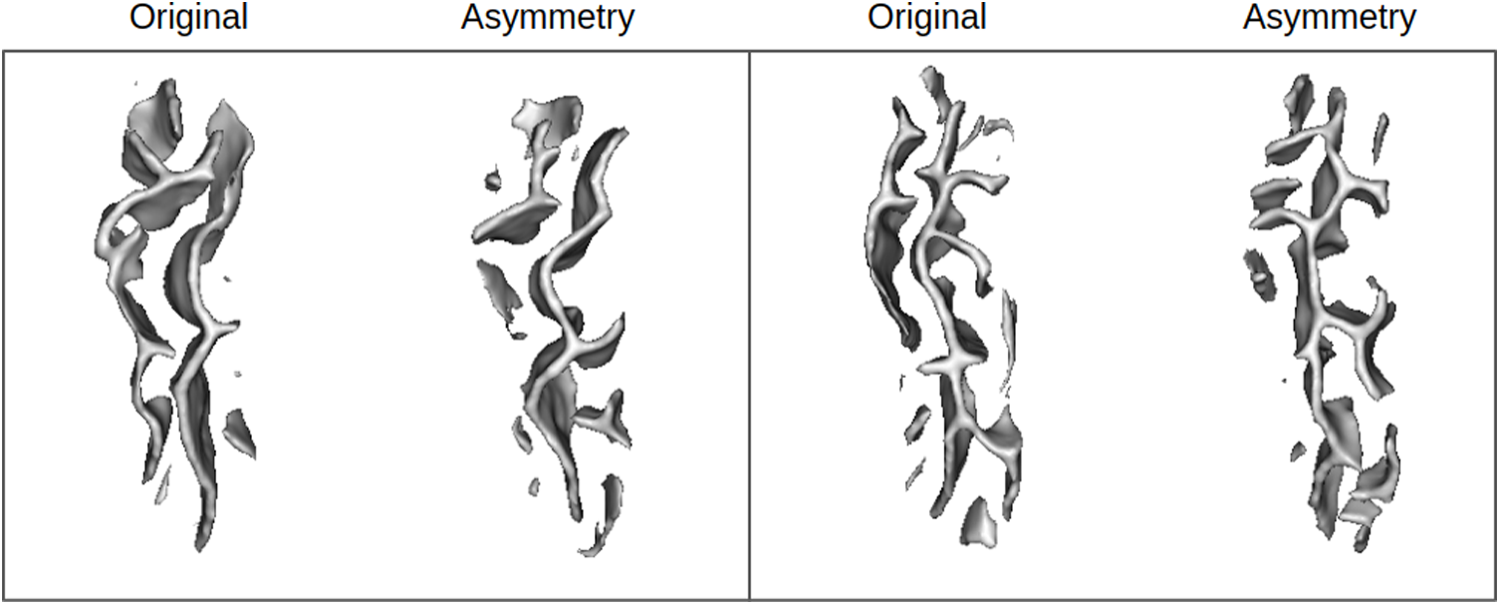
*Asymmetry benchmark.* Visualization of the original sulcal pattern and its flipped contralateral version for two subjects.

Our second benchmark leverages the asymmetries described in the central sulcus region which concern several folding features (Foubet et al., 2022; Sun et al., 2007; de Vareilles et al., 2022). Using crops of the left hemisphere as outliers enables to assess whether we can identify shape variations. In practice, this benchmark corresponds to the equivalent crop but in the left hemisphere. Left hemisphere distance maps are generated according to the same methodology as the right. Like our control crops of the right hemisphere, we computed a left central sulcus mask on the labeled dataset. To enforce the exact same crop size, we adapted the mask to match the adequate dimensions by adding or deleting a few voxels. Once the crops were obtained, they were flipped. During training, the right central sulcus mask was applied on the fly. We emphasize that we did not use the interhemispheric plane-symmetric coordinates but a mask specifically designed for the left central sulcus. This is especially important since there is a slight asymmetry in the position of the central sulcus between the two hemispheres (Davatzikos & Bryan, 2002). An example is presented in Figure 5.

### 3.5 Identifying outliers

Once the model has been trained on the controls, outliers identification can be performed at two levels. Traditionally, anomaly detection with AE is done based on the reconstruction error and an error map can be obtained comparing the input and the output (Pinaya et al., 2018; Schlegl et al., 2017, 2019). But one can also wonder about the distribution of outliers in the latent space. Are the outliers distributed differently? To answer this question, we investigated the detection power in the outliers’ distribution in the latent space and based on the reconstruction errors performed in the input space—which we call *folding space* in our case, as we study folding patterns. For both approaches, control test images and outlier images (deletion benchmarks, asymmetry benchmark and interrupted sulci) are encoded and reconstructed by our trained model.

#### 3.5.1 A specification on data

As mentioned in Section 3.3.2, our control test set comprises 200 subjects and the benchmarks are generated from the control test set. However, in order to prevent any form of data leakage, for each benchmark, the controls of the test set are different subjects from the altered subjects composing the benchmarks. For deletion benchmarks, since some subjects did not have any SS meeting the benchmark’s criteria, the number of subjects are different. Details for the different benchmark datasets are presented in Table 1.

**Table 1.** Number of subjects for the different benchmark datasets.

Deletion					
Benchmark dataset	200	500	700	1000	Asymmetry
N controls	90	34	54	75	100
N outliers	90	34	54	76	100

#### 3.5.2 On the latent space

Investigating the detection power in the latent space is not straightforward since our latent space may comprise many dimensions. Thus, we explore the latent space based on three steps. First, based on visualization; then, to assess whether relevant folding properties have been encoded, we train SVM classifiers on the latent space; last, we travel through the latent space to better understand it.

*A hint from the visualization.* For both of our benchmarks and the interrupted central sulci, we first sought to have a visualization of data distribution in the latent space. Therefore, we projected encoded data into a smaller space of two dimensions with the UMAP algorithm (McInnes et al., 2018, 2020). This projection enables us to get a first hint as to how outliers are represented.

*Assessing the detection power on the benchmarks.* However, the UMAP algorithm drastically reduces dimensions, leading to some information loss. We tried to assess whether relevant information regarding folding patterns was encoded in the latent space. Therefore, we trained linear SVM (Pedregosa et al., 2011) on the latent codes using 5-fold stratified cross-validation to classify between control data and benchmark. Performance is assessed based on the ROC curve.

*Quantifying the marginality of interrupted central sulci.* As interrupted central sulci are very few, we cannot use classification as we did for the benchmarks. Classic machine learning out-of-distribution algorithms are more suited. Therefore, to quantify whether the interrupted sulci are likely to be detected from their location in this reduced space, we applied two classic algorithms, One-Class SVM (OCSVM) (Pedregosa et al., 2011; Schölkopf et al., 2001) and isolation forest (Liu et al., 2008; Pedregosa et al., 2011), based on the data coordinates in the UMAP space.

*Traveling through the latent space.* Finally, to better understand the encoded properties and the learned representations, we leverage the generative power of the β−VAE. We computed average representations from different sets of data points, taking the mean for each dimension of the latent space. We then reconstructed these vectors. To further analyze the latent space, we traveled through it, going from one point, either the average pattern or a subject, to another point in the latent space, linearly interpolating vectors and reconstructing them.

#### 3.5.3 On the folding space

Outlier identification in the folding space relies on the model’s error. The reconstruction errors’ distributions were compared visually and assessed with the Kolmogorov-Smirnov test for the benchmarks and with the Mann-Whitney U-test for interrupted central sulci. For both cases, the null hypothesis was that the two distributions were identical.

One strength of analyzing this space rather than the latent space is that the model’s errors can help understand and locate the rare patterns’ characteristics. To localize the errors, we commonly look at the residuals, which are the difference between the input and the reconstruction of the model. This corresponds to what the model has missed or added. To differentiate these two types of errors, we looked at them independently, computing the difference between the input and the output, that is, the model’s omissions, and between the output and the input, that is, the model’s additions. It is particularly interesting in the case of interrupted sulci, as we could expect that the model makes them continuous.

### 3.6 Generalization to another region and validation

Our benchmarks act as a proof of concept on *controlled* outliers. To assess the interest of our method, we tested the reproducibility in another region and another dataset by transposing our methodology to subjects presenting isolated corpus callosum dysgenesis (CCD) for which an abnormal pattern has been described in the cingulate region. Finally, we validated the capacity of our method to distinguish patients from controls and to identify specific folding features of the patients, on subjects suffering from focal cortical dysplasia of type 2 (FCD2), which is a common cause of drug-resistant epilepsy.

#### 3.6.1 Children with CCD

The isolated corpus callosum dysgenesis (CCD) leads to a cortex anomaly located in the cingulate region. This disorder is a congenital malformation that results in a complete or partial absence of the corpus callosum. The corpus callosum is composed of fibers that connect the two hemispheres.

The dataset includes 7 children between 9 and 13 years old presenting an isolated CCD and 7 matched control children (Bénézit et al., 2015). For all children, the CCD was not associated with other malformations or developmental disorders. As presented before, we used T1-w MR images obtained from a Siemens Tim Trio 3 T scanner with an isotropic resolution of 1 mm.

The described anatomical anomalies associated with CCD include “sulci radiating on hemisphere medial surface, complete or partial absence of the callosomarginal sulcus and of the cingulate gyrus” (Bénézit et al., 2015). Therefore, we transposed our method to the cingulate sulcus region. Using the same methodology as presented before, we computed a mask of the cingulate sulcus (gathering the calloso-marginal anterior and posterior fissure in the BrainVISA nomenclature), resulting in crops of dimensions 30 x 128 x 125 and 30 x 130 x 108, which were padded up to 32 x 128 x 128 and 32 x 136 x 112 respectively for the right and left hemispheres. Since the corpus callosum connects the two hemispheres, CCD can be studied equally in both hemispheres. Therefore, we conducted our experiments in the right and in the left hemisphere.

#### 3.6.2 Patients suffering from FCD2

Focal cortical dysplasia of type 2 (FCD2) is a major source of drug-resistant epilepsy and is usually associated with anomalies of gyration and sulcation that may be localized in various areas of the brain. Specifically, in the central region, the Power Button Sign (PBS) has been proposed as a qualitative criterion to diagnose FCD2 (Mellerio et al., 2014). Nevertheless, the identification of this specific pattern was performed visually on a small cohort and has not been replicated since.

The dataset includes 19 controls and 29 patients who can have either a positive or a negative MRI (Mellerio et al., 2014). This distinction was proposed by Mellerio et al. (2012): a subject presents a positive MRI if “at least one of the cardinal MR signs of FCD2 (ie, cortical thickening, blurring, cortical and/or subcortical signal changes, transmantle sign) was present” (Mellerio et al., 2014). Twelve subjects have a negative MRI and 17 subjects a positive one in the dataset. In addition, the identified lesions may be located either in the right or in the left hemisphere, based on histological analyses. Table 2 provides the number of subjects of each group. For all subjects, we used the T1-w MR images. The majority was obtained with a GE Healthcare Signa 1.5 T scanner with a resolution of 0.98 x 0.98 x 1.40 mm. The other subjects were scanned with a 3 T scanner with a resolution of 1 x 1 x 1.2 (see Table 2).

**Table 2.** Characteristics of the dataset of patients suffering from FCD2.

Characteristics	ctrl	+ / right	+ / left	- / right	- / left
N	19	8	9	7	5
Age	31 (22-53)	21 (11-40)	34 (20-67)	16 (7-42)	18 (7-29)
N 1.5 T scanner	19	5	6	7	4

+ and - indicate whether the MRI is positive or negative. Right and left correspond to the side of the lesion. The age is given as average age (min-max).

The subjects were included in the original study because their epilepsy was localized in the central region. The consequence of the FCD2 in terms of folding patterns is still an area of research. According to Mellerio et al., patients demonstrate more side branches of the central sulcus and more often a PBS (Mellerio et al., 2014). The PBS is characterized by a precentral branch pointing between the central sulcus and one of its ascending branches. Hence, in this case, we worked on a region gathering both the central and the precentral sulcus of dimensions 78 x 86 x 99 in 1 mm resolution.

#### 3.6.3 Transposition of the method

For these two ROIs, we transposed the method in the same way. Specifically, we trained our β−VAE on the HCP dataset to model the inter-individual variability. We used the same data split as before. We used the hyperparameters obtained with the gridsearch on the central sulcus region for training. Choosing these parameters may lead to sub-optimal performances but enables us to have a first validation of our methodology. Analyses of the latent and the folding spaces are performed following the method described above for the central sulcus.

## 4 Results

### 4.1 Training results

Each training lasted for approximately 1 hour on an Nvidia Quadro RTX5000 GPU. We obtained with our gridsearch β = 2 and L = 75.

### 4.2 Assessment on synthetic known anomalies

#### 4.2.1 On the latent space

**Fig. 6.**
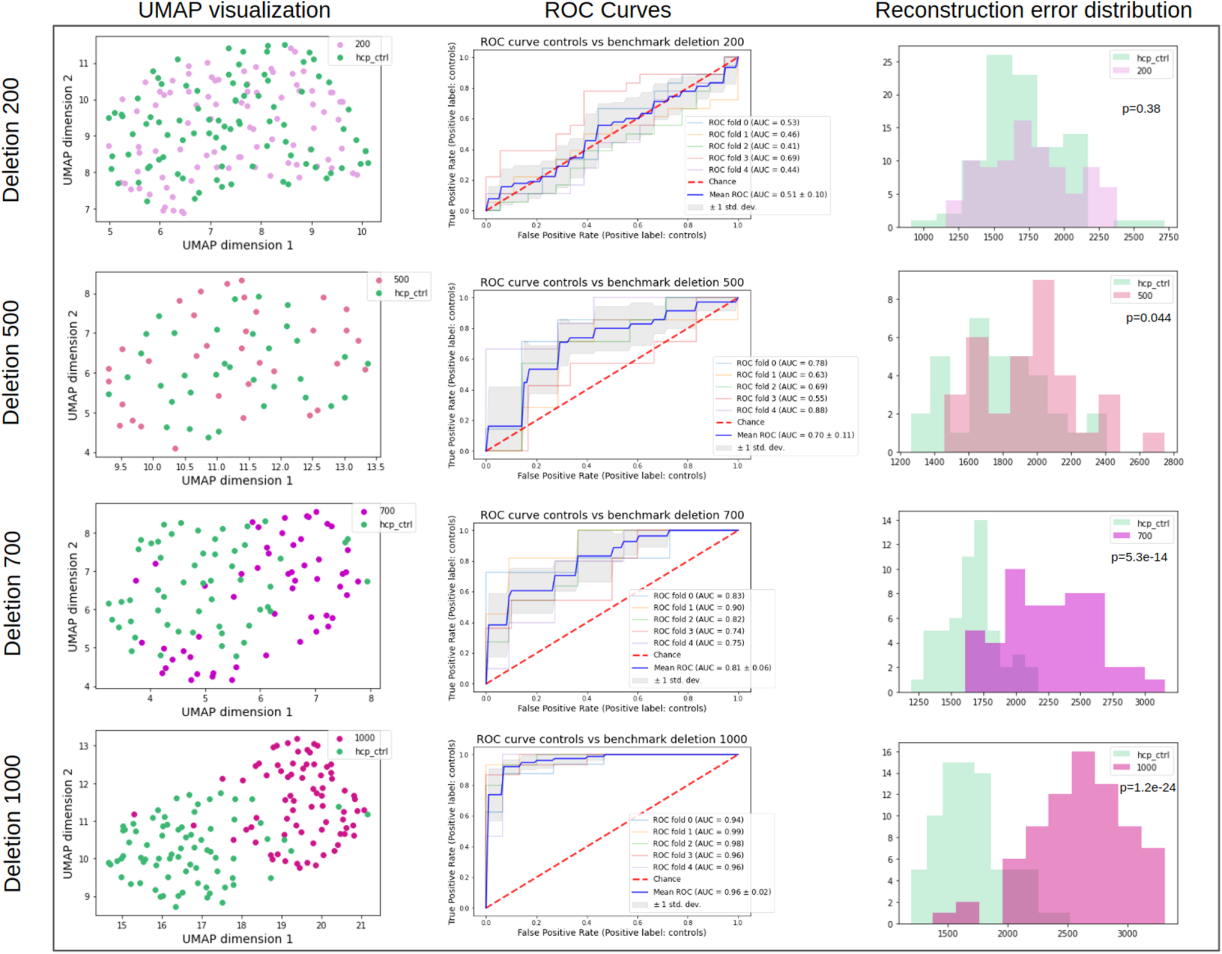
*Deletion benchmarks results.* For each row, controls are represented in green and benchmark data in pink. Left column: UMAP projection of benchmark and control data. Middle column: ROC curves of classification of control and benchmark data. Right column: reconstruction error distributions and p-value of the Kolmogorov-Smirnov test with the null hypothesis that the two samples come from the same distribution.

UMAP latent space visualizations for the four deletion benchmarks are presented in Figure 6. For the benchmark 200, benchmark data are rather homogeneously distributed among control data, suggesting that simple surfaces of sizes between 200 and 500 voxels are too subtle to be encoded differently. Indeed, as shown in Figure 4, small, simple surfaces can correspond to tiny branches that display a high variability in the population. The distribution of benchmark 500 seems to be not completely similar to the control’s, but the restricted number of subjects makes it hard to conclude. However, the trend becomes more pronounced for benchmarks 700 and 1000 where fake anomalies are gradually gathered and their distributions are different from the controls. These results are confirmed by the ROC curves (Fig. 6). Even when using all the latent dimensions, classification results are very poor for benchmark 200 (AUC = 0.51), supporting that the deleted branches may be too melted into the inter-individual variability. Classification performances are also very low for benchmark 500 (AUC = 0.70). They start to be slightly better for benchmark 700 (AUC = 0.81) but are very good only for benchmark 1000 (AUC = 0.96).

**Fig. 7.**
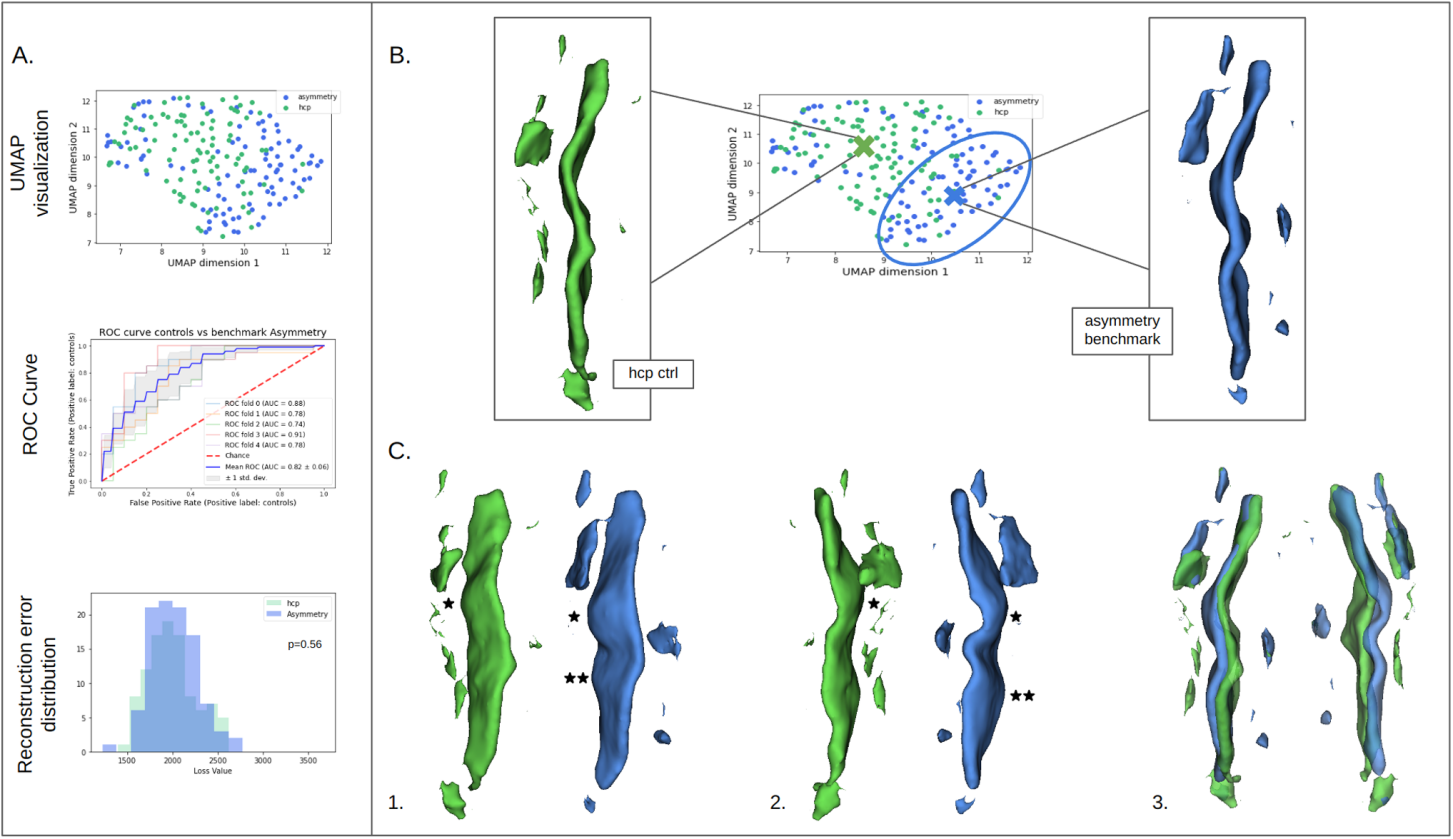
*Asymmetry benchmark results.* Controls are represented in green and benchmark data in blue. (A) UMAP projection of benchmark and control data, ROC curves of classification of control and benchmark data, and reconstruction error distributions. (B) Averages for the control subjects, that is, right hemispheres (in green), and for the highlighted asymmetry subjects, that is, left hemispheres (in blue). These averages are also placed on the UMAP dimensions. (C) 1. and 2. Respectively side and bottom views of the averages of B. The single star indicates a single-knob configuration, and the two stars indicate the second knob of a double-knob configuration. (C) 3. Superposition of the two averages respectively in upper and bottom view.

For the asymmetry benchmark, UMAP visualization demonstrates a good separation between the right and the left hemisphere (Fig. 7A), which is verified by the classification of the whole latent space (AUC = 0.82). These results suggest that specific shape features are encoded among other properties in the latent space.

To better understand the asymmetry characteristics encoded by the model, we leveraged the generative power of our β−VAE. Figure 7B and C show the average patterns for the right (green) and the left hemisphere (blue) as encoded by our model. The hand knob of the right central sulcus seems to be slightly higher and shallower than in the left hemisphere. Moreover, the double-knob configuration appears more prominent in the left hemisphere.

#### 4.2.2 On the folding space

We then investigated whether the folding space, that is, reconstruction errors, was relevant for identifying outliers. For deletion benchmarks, we observe a similar trend as in the latent space. For deletion 200, we cannot see a difference of distributions (p-value = 0.38). However, from deletion 500 we can see a stall with the deletion benchmarks having significantly higher reconstruction errors (p-values of 0.044, 5.3e-14 and 1.2e-24 for benchmarks 500, 700, and 1000 respectively) (Fig. 6). On the contrary, for the asymmetry benchmark, there is no significant difference, nor a trend, in the reconstruction error distributions (Fig. 7).

### 4.3 Application on the case of interrupted central sulcus

#### 4.3.1 On the latent space

**Fig. 8.**
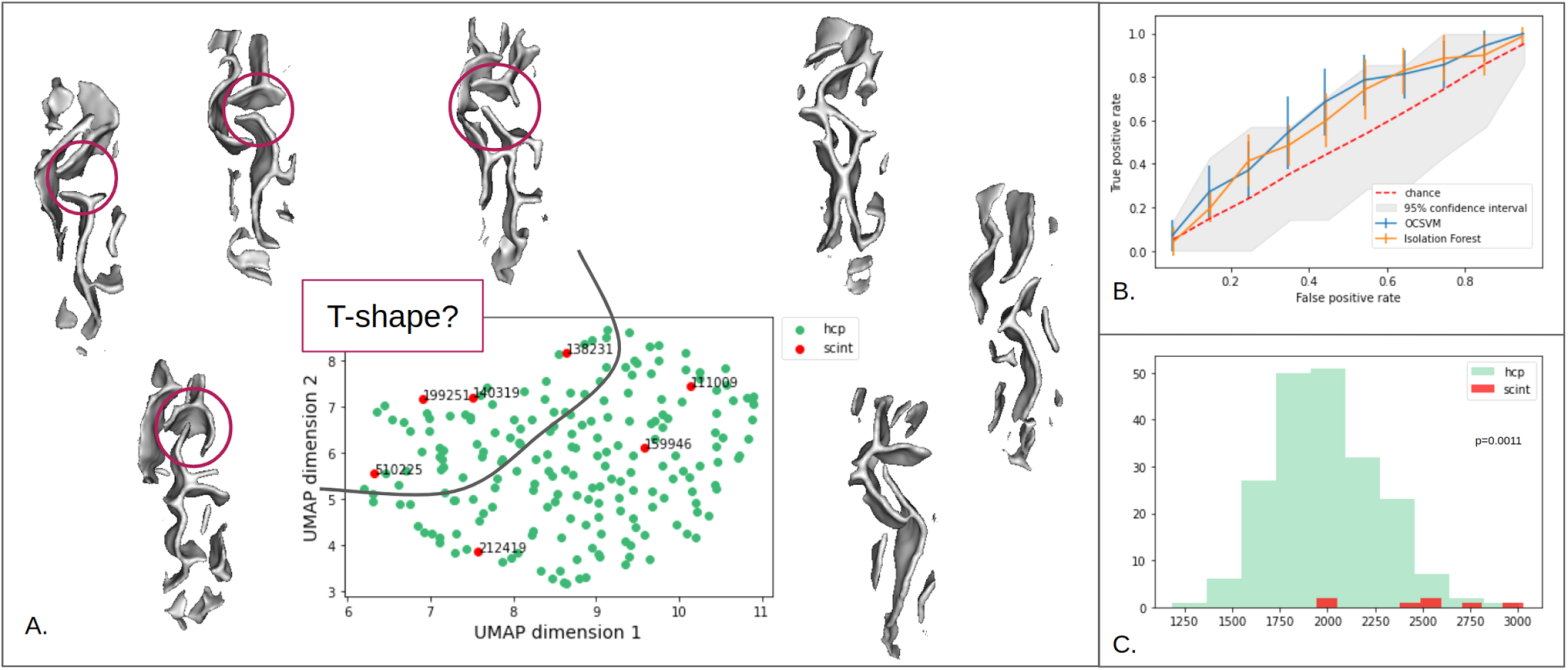
*Interrupted central sulci on UMAP space.* (A) Interrupted central sulci shape distribution in the UMAP space. The 3D folding patterns of the subjects are positioned according to their location in the UMAP space. For instance, the pattern located in the lower left corner corresponds to subject 510225 in the UMAP representation. Subjects with interrupted sulci on the upper left of the UMAP visualization seem to correspond to an interruption with a T-shape pattern. (B) Outlier detection performances using OCSVM and isolation forest on the interrupted CS. (C) Controls and interrupted CS reconstruction error distributions.

The UMAP projection from the latent space is shown in Figure 8A. On this distribution, most interrupted central sulci are at the margin of the point cloud except for one. Thus, it appears that the representation learned by our model enables to project rare patterns at the margin of the population. Interestingly, when we look at the pattern of each one of the interrupted sulci, it seems that a specific pattern, the “T-shape” pattern (Mangin et al., 2019) is specifically located on one side of the representation. Figure 8B shows the assessment of the marginality of the interrupted sulci based on an OCSVM and isolation forest. Error margins correspond to various UMAP projections, suggesting that the ability to detect interrupted CS in the UMAP space is very dependent on the UMAP projection. Interrupted CS detection is within the confidence interval, but the curves are close to the superior bound suggesting a tendency. However, interrupted CS positions in the UMAP space are not enough to detect them: detecting 5 interrupted CS out of 7 would lead to more than 40% of false positives. Nevertheless, some other patterns considered as controls and detected as outliers might also be rare.

#### 4.3.2 On the folding space

When analyzing the detection power on interrupted CS in the folding space, we first note that the reconstruction errors’ distributions seem to be different between HCP controls and interrupted CS (p-value = 0.0011). This result suggests that our model has more difficulties to reconstruct the input and that reconstruction error could constitute a relevant metric to detect rare or abnormal patterns. However, having only seven subjects strongly limits our conclusions and this should be replicated with more data.

**Fig. 9.**
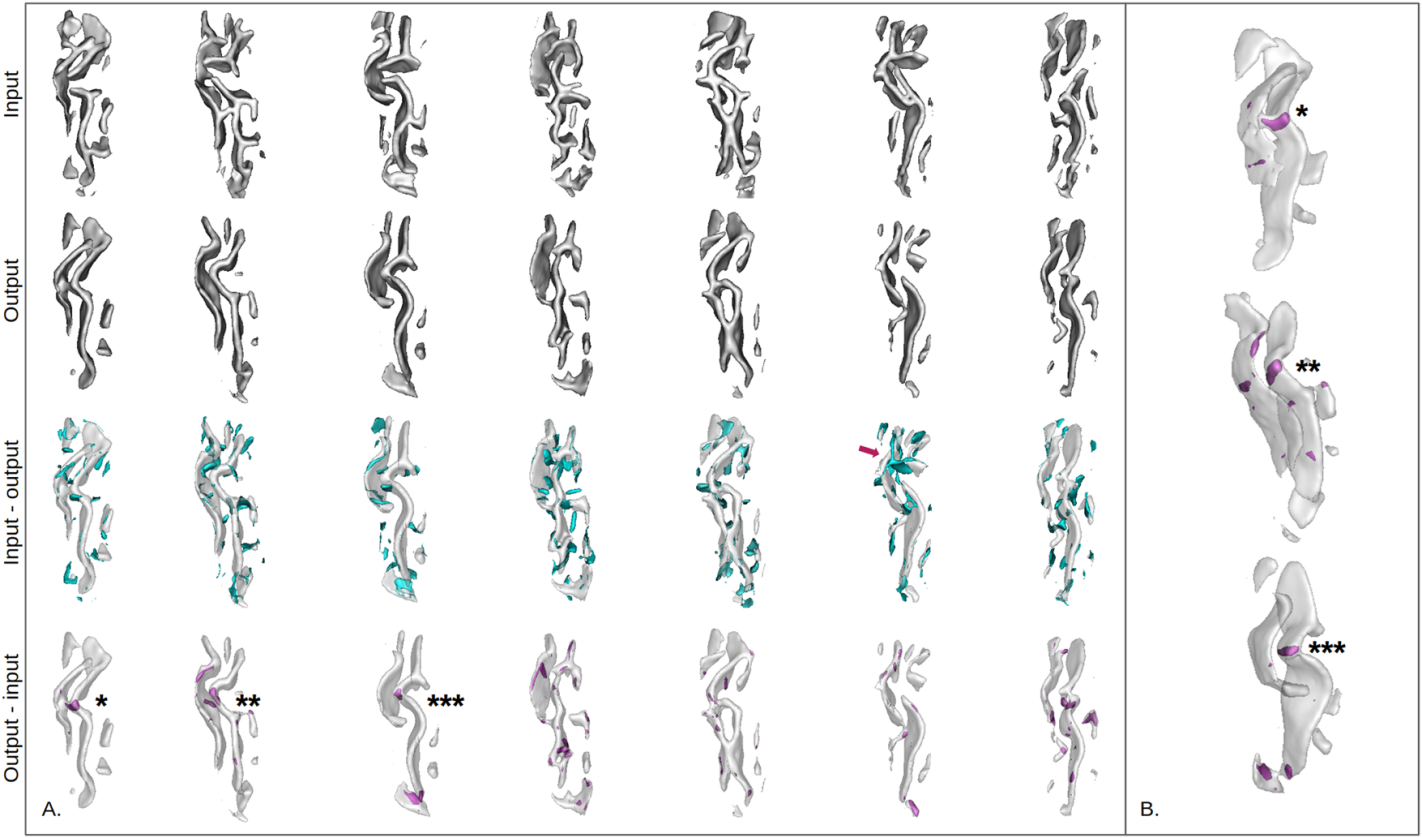
*Reconstructions and residuals for all seven interrupted sulci.* (A) For all rows, distance maps are converted to meshes for an easier visualization. First row: input data. Second row: reconstruction of the model. Third row: Reconstruction of the model with the difference between the input and the output, that is, the model’s omissions (in blue). The purple arrow highlights an omission corresponding to a perpendicular branch pointing toward the frontal cortex. Last row: Reconstruction of the model with the difference between the output and the input, that is, the model’s additions (in purple). (B) Rotated view of the reconstructions represented with asterisks in the last row of A. The asterisks show interruptions of the central sulcus which have been filled by the model.

Observing the reconstructions and the residual maps of Figure 9 gives clues into the way our model has encoded the interrupted CS. First, we can note that the reconstruction quality is quite good visually. The model’s omissions appear to be quite noisy (blue small fold pieces). The arrow points out an omission beyond noise which corresponds to a perpendicular branch pointing toward the frontal cortex. Such a pattern might be an uncharacteristic configuration. It is interesting to note that in six out of seven cases, the model transformed interrupted sulci into continuous patterns (“output-input” visualizations). Unlike the omissions, the model additions are rather localized. Moreover, the asterisks show where the model has filled the interrupted sulci. Such visualization could be useful to identify rare patterns like interruptions or perpendicular branches.

### 4.4 Application to corpus callosum dysgenesis

#### 4.4.1 On the latent space

**Fig. 10.**
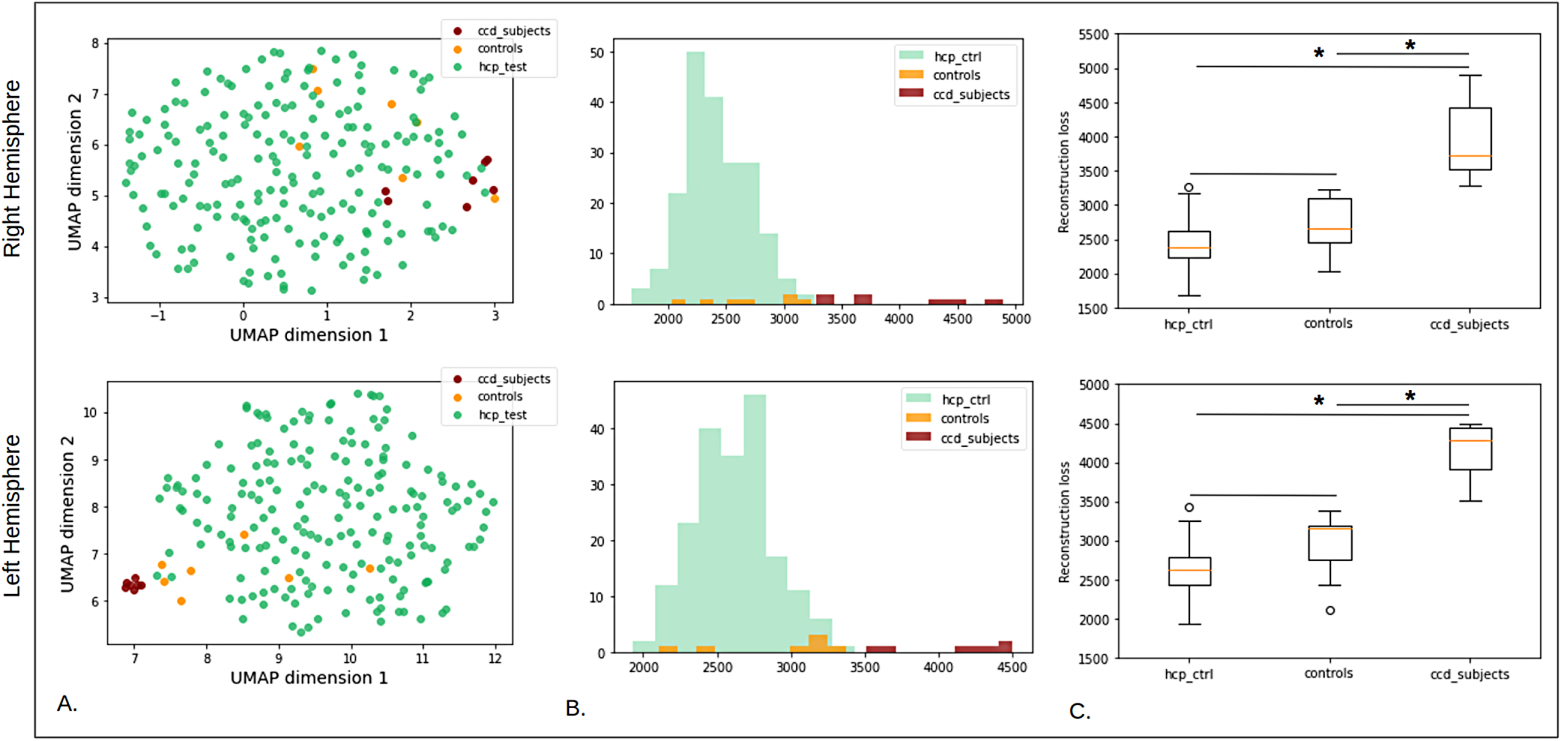
*Results on corpus callosum dysgenesis (CCD) subjects.* First row: right hemisphere. Bottom row: left hemisphere. For both rows: (A) UMAP projections of CCD subjects, control children, and HCP test. (B) Reconstruction error distributions for the CCD subjects, control children, and HCP test. (C) Reconstruction error variations for the CCD subjects, control children, and HCP test. Significant differences between populations according to the Mann-Whitney test are indicated with an asterisk.

We first compare distributions of CCD children (n = 7) with control children (n = 7) acquired in the same conditions and with HCP adult subjects (n = 200). UMAP projections, presented in Figure 10A, give different results depending on the hemisphere. For the right hemisphere, it seems that most children controls are included in the distribution of adult controls (hcp_test in green). Five out of the seven subjects having a CCD are located at the margin of the controls, suggesting that their latent representation differs from the average cingulate sulcus pattern. However, two subjects are in the middle of the controls. In the left hemisphere, only three control children are clearly in the control adult distribution. The other four are closer to the CCD subjects but they seem to be still distinct. Indeed, CCD subjects are gathered very close to each other. This could be due to the fact that there may be an age effect between children’s and adults’ brains or a site effect (different scanners, resolution), which we tried to reduce by using skeleton-based images but which may still remain. Nevertheless, we can still observe a difference in distribution between control children and CCD subjects.

#### 4.4.2 On the folding space

Regarding reconstruction error distributions (Fig. 10B), we observe for both hemispheres that control children seem to have the same distribution as adult controls, which is confirmed by Figure 10C. (p-value = 0.034 and 0.017 respectively for right and left hemisphere). On the contrary, CCD subjects present higher reconstruction errors that are significantly different from both HCP controls (p-value = 3.6e-06 for the two hemispheres) and children controls (p-value = 0.0011 for the two hemispheres). Therefore, it seems that there is a complete individual separability of the CCD patients which is very promising and should be replicated with more data.

**Fig. 11.**
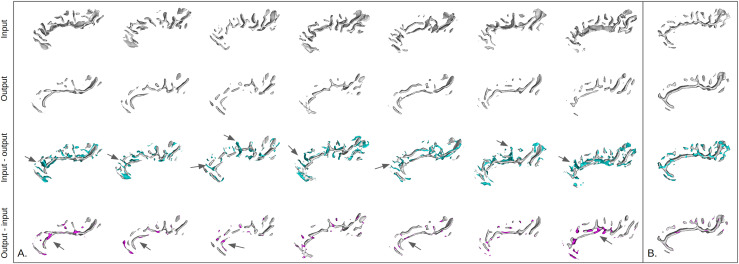
*Right cingulate sulcus reconstructions and residuals for the CCD subjects and one control.* (A) CCD subjects. (B) One control subject from the same cohort. For both (A) and (B): each column corresponds to a subject. For all rows, distance maps are converted to meshes for an easier visualization. First row: input data. Second row: reconstruction of the model. Third row: Reconstructions of the model with the difference between the input and the output, that is, the model’s omissions. Last row: Reconstructions of the model with the difference between the output and the input, that is, the model’s additions. The arrows highlight interesting features added or missed by the model.

The reconstructions presented in Figure 11 highlight the singularities of CCD. The model’s additions mostly make the cingulate more continuous than initially. The model’s omissions are mainly small branches perpendicular to the cingulate sulcus that are radially oriented.

### 4.5 Application to patients suffering from FCD2

#### 4.5.1 On the latent space

**Fig. 12.**
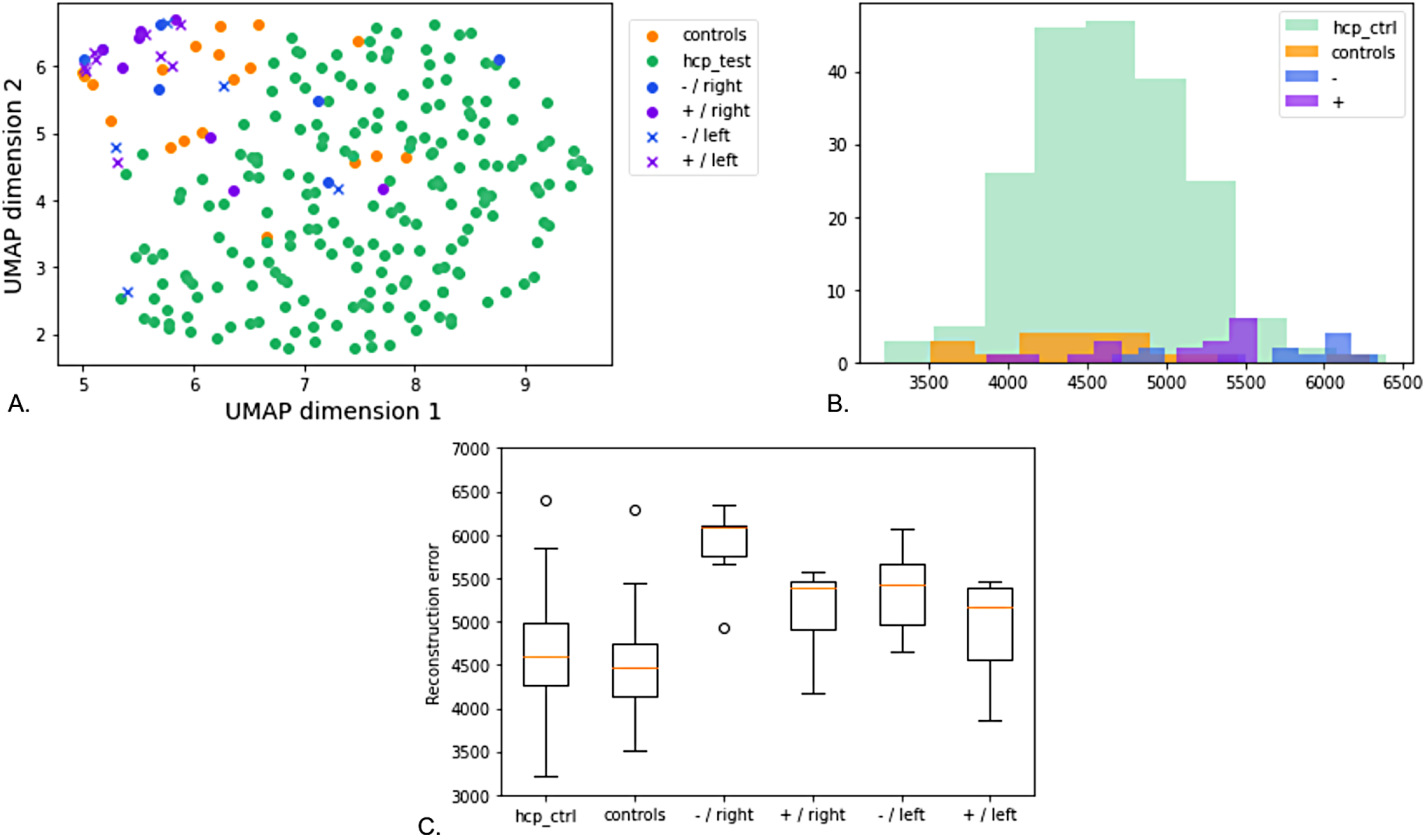
*Results on patients suffering from FCD2.* (A) UMAP projection of patients with a positive MRI (indigo), patients with a negative MRI (blue), controls of the same dataset (orange), and HCP test subjects (green). The crosses correspond to subjects with a lesion in the left hemisphere. (B) Reconstruction error distributions for the four groups (patients with negative or positive MRI, controls, and HCP subjects). (C) Reconstruction error variations for the different groups. Patients of each group have been separated depending on the lesion’s location.

Figure 12A presents the latent representation projected to the UMAP space of patients with positive MRI and lesion in the right or left hemisphere (respectively +/right and +/left), patients with a negative MRI and lesion in the right or left hemisphere (respectively -/right and -/left), and controls acquired in the same conditions and HCP subjects. We first notice that similar to what we observed with the CCD subjects, the controls do not have the same distribution as HCP controls: they are more located at the margin and seem to represent a transition towards the patients. For patients with positive MRI, those with lesions in the left hemisphere (represented with crosses) do not appear to be distributed like controls. 5 out of the 8 patients with positive MRI and right lesion are at the complete margin of the point cloud and beyond the controls of the same database. -/right subjects seem to be equally projected at the margin or in the HCP distribution. Last, with the exception of one subject, the projection of -/left patients is quite similar to that of controls.

**Fig. 13.**
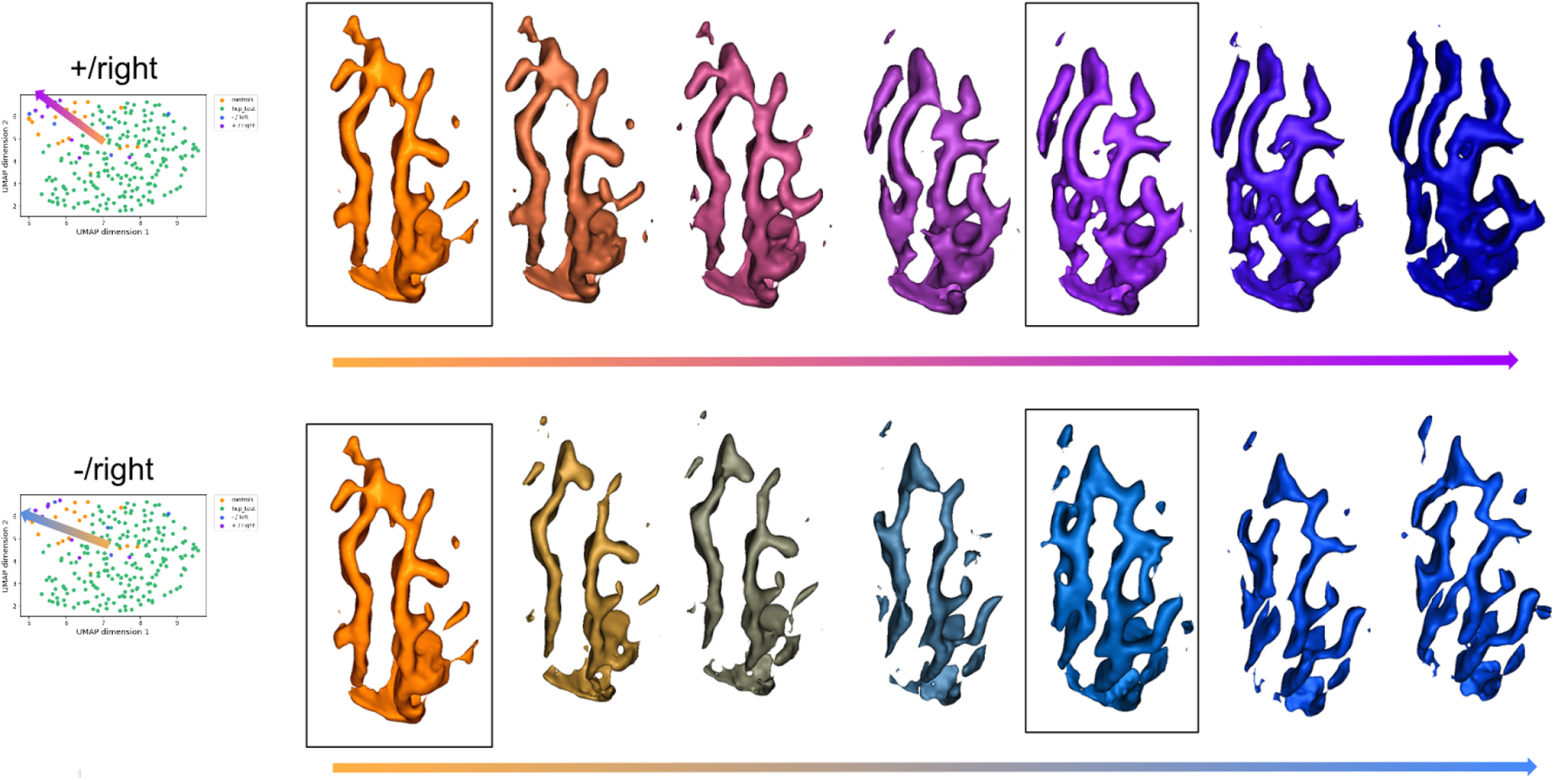
*Traveling through the latent space from the control centroid to the centroid of each group of patients and beyond.* The insets are the centroid-generated patterns. We travel through the latent space from the control centroid to each patient group centroid and beyond as illustrated by the arrows. The intermediate patterns between the insets correspond to interpolations along the arrows. First row: From controls to +/right patients. Second row: From controls to -/right patients. Only patients with lesions located in the right hemisphere were used to generate the averages. Colors match Figure 12: controls are represented in orange, patients with positive MRI in indigo, and patients with negative MRI in blue.

To try to decipher the pattern characteristics of the two groups of patients with the lesion in the right hemisphere, we generated the average patterns. The insets of Figure 13 show the three average patterns for the patients having a positive MRI (indigo), the patients with a negative MRI (blue), and the associated controls (orange). The control pattern is composed of the central sulcus (on the left side) and of the precentral sulcus (on the right side). Like the average pattern based on HCP test subjects, the central sulcus of these control subjects is composed of a knob. The precentral sulcus seems rather continuous. The pattern of patients with a positive MRI presents a central sulcus with a wider knob. In addition, we can observe a branch that links the central sulcus to the precentral. Last, the pattern of patients with a negative MRI (blue) shows a central sulcus with a quite small knob. Unlike the control precentral which is quite flat, the precentral of both groups of patients seems curvier, especially for the patients with a negative MRI.

For both groups, we then traveled along the axis from the control centroid to each patient group centroid and beyond (as represented by the arrows). Samples between the insets are linear interpolations which are then reconstructed. The furthest samples are likely to be unrealistic as they were sampled from less represented areas. Going from the control centroid towards the patients with positive MRI, we see the hand knob progressively widening and its bottom part merging with the precentral sulcus. Interestingly, the average pattern of the +/right subjects may even suggest an intermediate PBS pattern. Interpolations beyond are more complex to analyse and seem to present merged central and precentral sulci.

From the control centroid to the patients with a negative MRI, the central sulcus seems to flatten and present a very slight knob in the average pattern. The precentral slightly curves and shows more branches. Beyond the average of -/right patients, the interpolations display unlikely configurations with a CS presenting several interruptions and a long precentral with several branches.

Nevertheless, the results must be taken with caution since we only have a small number of subjects. Future work will need to be replicated with a larger dataset to validate these findings.

#### 4.5.2 On the folding space

Regarding the reconstruction error, contrary to CCD subjects, all the samples from the cohort are included in the range of HCP subjects (Fig. 12B). This could be due to the lower resolution of most subjects. However, the distribution of both groups of patients seems to be different from that of the controls (Fig. 12B and C). In Figure 12B, all patients with negative or positive MRI were considered, whether their lesion is located in the left or in the right hemisphere. Figure 12C. presents the details for each group. Both figures suggest that patients with negative MRI have higher reconstruction errors and thus are more difficult to reconstruct. This is confirmed by the p-values reported in Table 3. It is all the more interesting as these are the patients whose MRI shows no signs of FCD2. We do not observe a difference in the distributions between HCP subjects and controls (adjusted p-value = 0.41). -/right patients are the only group with a distribution significantly different from that of the controls (adjusted p-value = 0.0020). For +/right patients, it seems that their reconstruction errors tend to have a different distribution but it is not significant. It is interesting to note that although patients with a lesion located in the left hemisphere present fewer differences with the controls than patients with a lesion in the right hemisphere, they are still not similar to controls. A potential explanation is that the events perturbing the neuro-development and resulting in a lesion in the left hemisphere may have had other consequences, less marked, in other areas of the brain. Nevertheless, as we deal with only a small number of patients, this should be replicated with more data in order to conclude.

**Table 3.** Significativity of Mann-Whitney U test.

Groups	p-value	Adjusted p-value
HCP vs controls	0.083	0.41
controls vs +/right	0.014	0.069
controls vs -/right	**0.00040**	**0.0020**
controls vs +/left	0.025	0.12
controls vs -/left	0.0095	0.047

The null hypothesis is that two samples have the same distribution. Adjusted p-values were corrected with the Bonferroni method. Bold indicates significant at threshold 0.005.

**Fig. 14.**
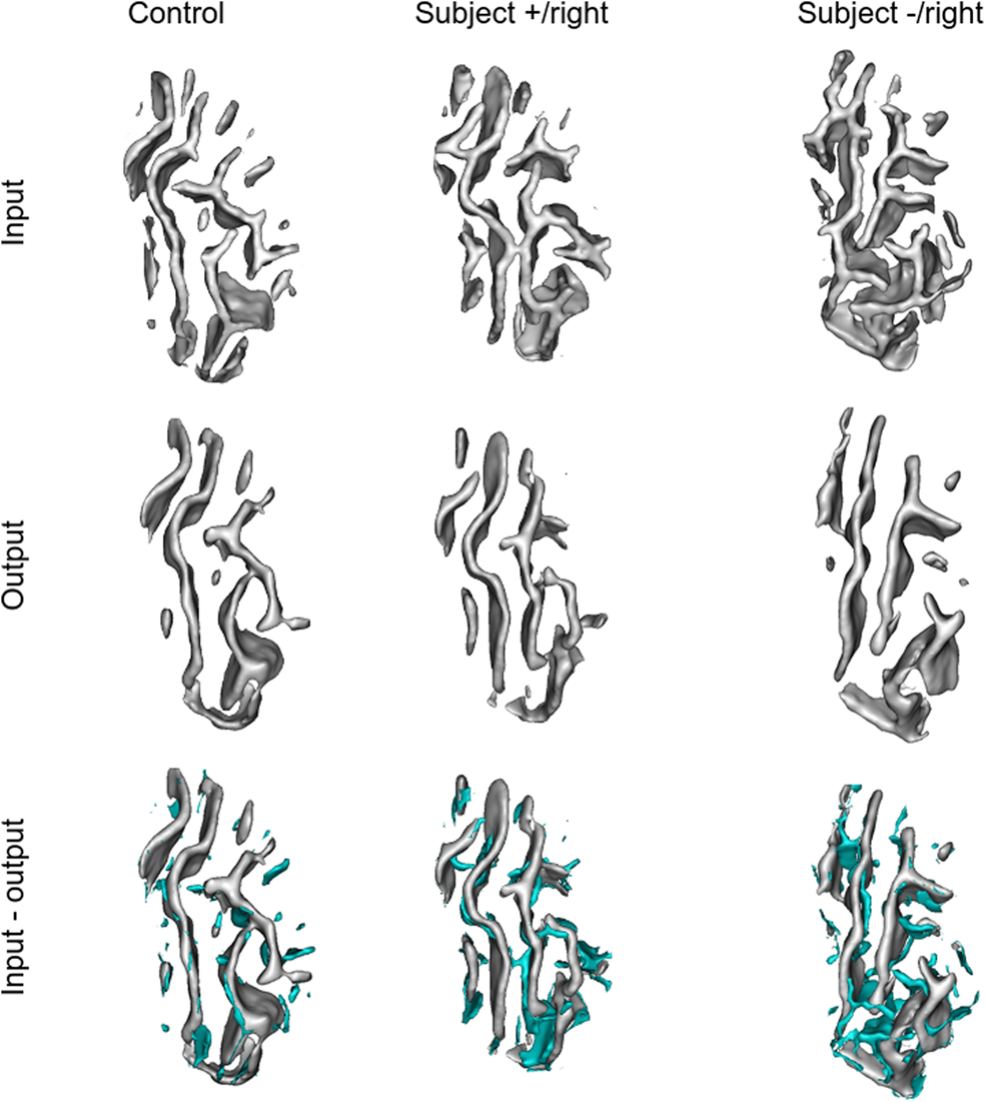
*Right central region reconstructions and residuals for the patients suffering from FCD2 and controls.* Each column corresponds to a subject from each group (control, patient with a positive MRI, patient with a negative MRI). For all rows, distance maps are converted to meshes for easier visualization. First row: input data. Second row: reconstruction of the model. Third row: Reconstructions of the model with the difference between the input and the output, that is, the model’s omissions. Note that all patients represented have the lesion located in the right hemisphere.

Figure 14 presents the reconstructions for a control subject and patients with positive and negative MRI (and a lesion located in the right hemisphere). Additional subjects of the different groups are presented in the Annex 8 of the Supplementary Information. Contrary to the previous results, there is no row for the model’s addition (output-input) because no error was important enough to appear.

First, we can notice for the control that despite being a simplified version of the input, the reconstruction is quite good.

For the patient with a positive MRI, the model’s omissions include small branches. However, one important feature is also the connection between the central and the precentral sulci which is missing. Therefore, a connection between these two sulci may be a rare feature. This could be confirmed by Figure 9A where a perpendicular branch from the CS to the precentral was missing in the reconstruction.

The reconstruction of the patient with a negative MRI visually confirms the results of Figure 12: the reconstruction quality is lower. We can notice that the subject presents a PBS that the model cannot correctly reconstruct.

## 5 Discussion

This work proposed a methodology to study rare folding patterns which was applied to the central sulcus region and to a described rare pattern, interrupted central sulci. Specifically, we represented folding patterns with distance maps and leveraged the generative power of the β−VAE to have a better understanding of the learned representations. In addition, we proposed a way to study the granularity of deviations that can be identified. We also compared the identification power of both the latent space and the folding space. Finally, we assessed the generalization of our methodology on a developmental anomaly in another region and the validation of our framework on a cohort of patients suffering from FCD2 led to promising results.

### 5.1 Latent space and folding space, two complementary information

In many anomaly detection works applied to medical images, the detection is performed based on the reconstruction error rather than in the latent space (Baur et al., 2020; Behrendt et al., 2022; Schlegl et al., 2019; Tschuchnig & Gadermayr, 2022). However, both of these spaces have their interest and could bring complementary information. In our work, we studied four types of rare patterns, two synthetic types, deletion and asymmetry benchmarks, and two actual rare patterns. These four categories differ from control data by their own characteristics and thus help to study the granularity detected, that is to say, the typology of rare features that can be identified. For instance, the asymmetry benchmark includes more double-knob configurations. Depending on the size of the deleted simple surface, deletion benchmarks represent different features: benchmarks 200 and 500 represent mainly a missing branch which may represent the normal variability of branches. Benchmark 700 could look like an interrupted sulcus in some cases or in others, like benchmark 1000, an unlikely configuration. Interrupted central sulci present a clear interruption and a rare arrangement of the shapes forming the central sulcus. Last, CCD subjects demonstrate a missing sulcus or missing sulcal parts and branches with different orientations. For patients suffering from FCD2, although PBS has been described, it is not present in all patients and is not specific; so in this case, we do not know whether there is a rare pattern and if so, what the pattern would be.

These different kinds of known deviations from the norm provide clues to the characteristics of rare patterns that can be identified respectively in the latent space or in the folding space of our model. As a matter of fact, the identification performances in the latent and in the folding space vary depending on the kind of patterns. For deletion benchmarks, the folding space, based on the reconstruction error, seems to enable the identification of unusual patterns from smaller modifications. Likewise, for the interrupted central sulci and the CCD subjects, despite the small number of samples, their detection seems to be easier on the basis of reconstruction error than in the latent space. In return, the error distribution of the asymmetry benchmark is not different from that of the controls, but the benchmark is well detected in the latent space. Therefore, the latent space could be more sensitive to shape arrangements than the folding space. The lack of difference in the error distributions may be due to the fact that the voxel-to-voxel differences between the right and left central sulci are local and subtle and could be embedded in the normal variability. In addition, the reconstruction error is for the entire image. Therefore, in the case of small and very local deviations from the norm, the reconstruction error alone is likely to be insufficient. A way to limit such effects could be to use a more local error, applied to sub-regions or patches for instance.

The difference in the outlier detection performance may also lie in the way our model encodes the outliers. Based on our results, we can consider several cases. First, a rare configuration is represented by several samples present in the training set. This would be the case with the asymmetry benchmark. Indeed, there are more double-knob configurations in the left hemisphere but single- and double-knob patterns coexist on both sides. In such a case, the distribution support of the left and right hemispheres is the same, but the densities differ, which could lead to a projection of the outlier at the margin of the latent space but to a good reconstruction. Second, the rare configuration is almost never represented in the training set and the model has not detected and thus encoded its local specificity. Then, the subject would be encoded with a “default” representation and projected in the middle of the other subjects. This would be consistent with the results of Guillon et al. (2021), where major anomalies (different parts of the brain from the one considered in the train set) were projected in the middle of the point cloud and reconstructed as the average reconstruction. It could be the case of the benchmark deletion 500 and of the interrupted central sulcus that is projected in the point cloud. Last, the outlier configuration is almost never represented in the training set but the model has detected the rare characteristic. The subject is then projected at the margin of the point cloud and the decoder has not learned this part of the latent space leading to a poor reconstruction (interrupted central sulci, CCD subjects).

Nevertheless, in all cases, a strength of the folding space is the possibility to localize the reconstruction errors and, in some cases, the unusual features. If not too noisy, reconstruction errors can be very informative. For example, in the case of interrupted central sulci, looking at the model’s addition permits clearly localizing what is atypical in a subject (Fig. 9). Similarly, in the case of the CCD subjects, the reconstruction errors highlight the presence of radial small branches that are typical of this brain disorder (Bénézit et al., 2015). The reconstructions of patients suffering from FCD2 may suggest that a connection between the central and the precentral sulci could be a rare pattern. But some noise remains, and it might be interesting to add an additional constraint to represent only errors that correspond to a minimum number of contiguous voxels. This could lead to a good explanation of the abnormality which is of major importance in the field and especially when applied to medical images. Other explanation methods exist, directly on the network such as Grad-CAM (Selvaraju et al., 2020) or on an OC-SVM applied on the learned features (Sohn et al., 2022) for instance; but the use of the reconstruction error is immediate and easy to implement. Hence, the latent space and the folding space, based on reconstruction error, can provide complementary information and both can be used to identify rare patterns.

### 5.2 Data size limitations and unknown number of rare patterns

The method should be further qualified because of the low number of our examples of rare patterns. While the study of a known rare pattern is interesting and important, having only seven samples severely limits our conclusions. Similarly, the poor results of benchmarks 500 and 700 in the latent space could be due to their small size, and having larger benchmark datasets could lead to increased performances.

Also, we assessed our method in the CS area on the benchmarks and on an existing rare pattern, but because few rare patterns have been described in this region, there may be other rare configurations in what we consider the control population. For instance, three morphologic variants in the central sulcus region have been introduced, representing 2.9%, 7.0%, and 1.8% of the studied population, opposed to 78.2% of “omega” shape, that is, the central sulcus knob and 10.1% of “epsilon” shape which corresponds to the double-knob configuration (Caulo et al., 2007). This multiplication of rare patterns in the population would make the identification of interrupted central sulci more difficult.

### 5.3 Dataset limitations

We just mentioned the size of the dataset but other considerations regarding our dataset should be acknowledged. First, for training, we used only one cohort, HCP. When our analyses are performed on the same cohort, this has the advantage of not being confronted with the site effect. However, when we apply our framework to other cohorts such as the children with CCD or the patients suffering from FCD2, we observe that the distribution of the cohorts’ controls is different from the HCP controls. In order to limit this effect, we could try to incorporate controls from other databases in the training.

Moreover, the HCP dataset has some particularities since some subjects are related or even twins. Yet, folding patterns have been shown to be partly heritable (Im et al., 2011; Pizzagalli et al., 2020). In this work, we did not apply a specific methodology to deal with this specificity and we hypothesized that the folding proximity of twins could be embedded in the inter-individual variability. Nevertheless, to validate this hypothesis, we should replicate our work with another cohort without twins, such as UK BioBank.

### 5.4 Relevance of synthetic benchmarks

Moreover, we can wonder about the relevance of our synthetic benchmarks. Although synthetic rare patterns are of high interest as they enable to quantify the performances on different degrees of deviations from the norm, a few works have been interested in them to our knowledge (Guillon et al., 2021; Meissen et al., 2022). But the use of fake deviations raises the question: do they constitute adequate rare patterns? A few studies introduced rare folding patterns based on the arrangement of their shapes such as the PBS (Mellerio et al., 2014), an interrupted central sulcus (Mangin et al., 2019), or a flat central sulcus (Sun et al., 2017). Here, we emphasize their advantage in the study of our understanding of the brain: they are evidence of neurodevelopmental processes and then stable throughout life (Cachia et al., 2021; Dubois et al., 2008). But other abnormal sulcal features have been studied and correlated with neurodevelopmental disorders, such as the depth, which demonstrated anomalies in autism spectrum disorder (Dierker et al., 2015; Nordahl et al., 2007) or Williams syndrome (Essen et al., 2006) for instance. Despite being another subject of study, a benchmark corresponding to central sulcus depth variations could be interesting to assess whether our framework can be extended to detect such anomalies.

Regarding our deletion benchmark, there may be several categories of deletion deviations. Some may be minor, as a small SS representing a tiny branch. On the opposite, some can correspond to a depth change, representing the presence of a pli de passage, and thus lead to an interrupted central sulcus which would be expected to be a major feature of the topology. Hence, our criterion, only based on the size of the SS may be insufficient and it could be interesting to others, such as topological criteria. In addition, the generation process of our benchmarks could introduce some biases. First, for the asymmetry benchmark, the size of the mask was slightly different for the right and the left hemisphere. We adapted the left mask to match the right one. Although it concerned only a few voxels and no major differences were noticeable, this could introduce a bias. For the deletion benchmarks, we did not ensure that the deleted simple surfaces were equally covering the different parts of the crop (central sulcus, small branches, etc).

In any case, having an unusual feature that can be incrementally increased, or comparing several types of features, helps characterize the detection power of a model and the features likely to be detected.

### 5.5 Learning relevant representations

When dealing with sulcal patterns and their high complexity, it may be easier to use representations of the folding which attempt to gather several subjects with similar patterns. Local averages of sulci, also called moving averages, enable to concentrate on the main features of the different patterns and are thus very useful to analyze folding patterns (Foubet et al., 2022; Guillon et al., 2022; Sun et al., 2012; de Vareilles et al., 2022). From a graph-based representation of the sulci, the identification of patterns can be done after computing similarity and applying a clustering (Meng et al., 2018). Our approach proposes another method to learn sulcal representations. From our cropped distance maps, the β−VAE learns a mapping to a latent representation which can then be reconstructed. Therefore, rather than explicitly computing pairwise similarity between the subjects, gathering them, and then analyzing the patterns, we hope that our β−VAE directly learns shapes that can be combined and arranged in patterns. The representations learned by our model seem to be relevant and consistent with some morphological characteristics of the central sulcus area.

First, the reconstruction of the average representation of the right central sulcus is composed of an upper knob whereas the left average tends to more towards a double-knob configuration (respectively green and blue sulci in Fig. 7B). This is one of the main known asymmetries in terms of patterns and it appears early in the development. It has been detected in infants of 30 weeks postmenstrual age (de Vareilles et al., 2022) and in adults (Sun et al., 2012).

We also observed differences in terms of curvature of the hand knob, with a hand knob more pronounced in the left hemisphere than in the right (Fig. 7B). Considering that we study a right-handed population, this could be related to handedness. With the lateralization of the hand motricity, we expect the motor area of the right hand in the left hemisphere and particularly the precentral gyrus to be more developed for right-handed subjects, pushing backward the upper part of the central sulcus which would result in a knob more pronounced. This interpretation is consistent with a study on one-handed subjects that showed that subjects born without a hand had a flatter central sulcus contralateral to the missing hand (Sun et al., 2017).

### 5.6 Generative power of β − VAE and comparison with other strategies

Since our proposed framework is able to encode relevant features regarding folding patterns, the generative power of the β−VAE can be exploited. Indeed, reconstructions and interpolations are tools to understand the folding variability. We have just mentioned that the learned patterns were relevant and consistent with those obtained by other methods, but our method has the advantage of being able to reconstruct and interpolate.

Nevertheless, other deep learning models could be interesting to study folding patterns. For instance, β−VAE reconstructions are known to be blurry contrary to GAN’s. Currently, this shortcoming is limited as we seek to have a simpler representation of folding patterns; still, for more subtle details, another model may be better suited. In addition, in the anomaly detection field, models that add constraints on controls distribution are quite appealing. For instance, deep One-Class Classification and its derivatives have been proposed to push control data into the smallest hypersphere in the latent space (Ruff et al., 2018). This could help increase the detection performance in the latent space. Nevertheless, no matter the architecture or the framework, an important limit to understanding what our model has really encoded is the high number of latent dimensions.

To better interpret and quantify deviations in the different latent dimensions, approaches have proposed to combine auto-encoders and normative modeling (Kumar et al., 2022; Lawry Aguila et al., 2022). Indeed, for some years, normative modeling has become very popular to investigate brain disorders, proposing a framework to study individual deviations of diverse variables from population-level trajectories (Marquand et al., 2019; Rutherford et al., 2022). It has proved to be particularly useful for various disorders, including psychiatric disorders (Rutherford et al., 2023). Such models could be interesting for our task as they would enable to obtain a quantification of the deviation. Nevertheless, the preprocessing of the data in order to obtain relevant folding features is not straightforward. In order to obtain automatic image-derived features, some recent works have proposed to combine VAE with a normative model in order to predict alzheimer disease (Lawry Aguila et al., 2022). The developed approach seems particularly interesting as the authors apply the derived normative modeling approach in the latent space, enabling to propose a quantitative metric of deviations for each latent variable (Lawry Aguila et al., 2022). Normative modeling and approaches combining it with deep learning could be a promising strategy to explore in the future.

One can also wonder about the representation of the folding patterns. In the introduction, we mentioned two main strategies: clustering and manifold. Usually, these two approaches are applied to a continuous space. Nevertheless, if we consider sulcal shapes as symbolic entities that can be combined and arranged, we could represent folding patterns based on a discrete space rather than a continuous one. As such, VQ-VAE (van den Oord et al., 2017) seems to be an interesting representation to compare with our present results.

### 5.7 Sensitivity to outliers

Finally, this framework of outlier detection based on training on control subjects alone may be sensitive to outliers present in the training set. Having a contaminated dataset could severely limit the detection performances, at least in the folding space which is based on the reconstruction error. It has been reported in a brain tumor detection problem that having 3% of outliers in the training set (about 1000 samples) leads to a decrease of 5% of the AUROC and to a 13% decrease if the contamination reaches 12% of the training set (Behrendt et al., 2022). Therefore, one serious shortcoming of our paradigm is that we do not know the outliers we are looking for. Applying our framework to a control population alone in order to bring out rare patterns may limit the different patterns that can be identified. A way to tackle this issue and to increase the patterns detected would be to exploit the presence of outliers in the training set as proposed in Qiu et al. (2022). In their technique, the authors introduce an iterative joint training where they assign labels (anomalous or control) to the examples, and then optimize the network’s parameters to better identify the anomalies. Such a method could also enable to project the outliers more at the margin of the latent space. The impact of the presence of outliers during training on the latent space has not yet been investigated to our knowledge. If, as we suggested before, outliers present in the training phase are encoded at the margin of the distribution, that is, in a different area of the latent space, it could be interesting to deepen our analysis, based on clustering for instance.

### 5.8 Generalization of the approach: towards an analysis of the whole brain?

This work has shown that our approach had successfully encoded some relevant features of the folding patterns in the central sulcus region but it is attractive to think about the behavior and results we could obtain in other parts of the brain. Here, we assessed the generalizability and validated our framework on two other datasets and other regions. Our results suggest that our method can well transpose in other brain regions. Specifically, even if we use the hyperparameters (β and L) optimized for another area, the learned representations still enable us to distinguish between control and outlier subjects. This is all the more interesting that in the case of the CCD subjects, the two studied regions are rather different. The central sulcus is one of the first folds to form and is rather stable, contrary to the cingulate region that is more variable (Sun et al., 2009). Therefore, it seems that no matter the folding variability of the zone, our framework can be applied. This encouraging result raises a question regarding the procedure to adopt to extend our analysis to the whole brain. A way could be to define a set of regions, consistent with the cytoarchitecture and function and to train our β−VAE on each region. In particular, some areas seem to be interesting from a clinical point of view (Borst et al., 2014; Gervais et al., 2004; Hotier et al., 2017; Provost et al., 2003; Yücel et al., 2003). Our future works may thus focus on proposing an adequate methodology to tackle the whole brain.

On the other hand, when we applied this framework to CCD subjects and patients suffering from FCD2, we also operated a domain shift. Indeed, for the CCD subjects, the dataset to explore included exclusively children while the β−VAE was trained on young adults. Despite folding patterns being reported as trait features (Cachia et al., 2016), such an age variation may have an impact. In addition, beyond dealing with children, the site and the scanner are different. Similarly, in the case of patients with FCD2, the subjects were not homogeneous in terms of age and scanners. Such differences have been reported to affect the generalizability and the performances on various targeted tasks. In terms of distributions in the latent space, despite the fact that the distribution of the controls does not seem to completely overlap the distribution of the HCP controls, the patients still seem to present a different distribution than both controls’ populations. Moreover, the domain shift does not seem to have an effect on the folding space where controls reconstruction errors are not significantly different from HCP contrary to CCD subjects that have significantly higher reconstruction errors. The results of patients suffering from FCD2 are more complex to interpret as the consequence, in terms of folding, remains unclear. Still, the reconstruction errors of the controls are similar to HCP subjects and patients seem to have higher reconstruction errors. It is particularly interesting for -/right patients as their MRI does not present visual signs of FCD2 and could thus help in their identification and in the localization of the brain region involved. However, having only fewer than a dozen subjects makes it difficult to conclude and we will continue to explore these questions in further studies and replicate our results with larger datasets.

To conclude, this study proposed a framework to identify rare and abnormal folding patterns based on the modeling of the inter-individual variability. With a new representation of folding patterns, we proposed a model that was able to encode relevant folding characteristics. The use of synthetic rare patterns enlightened the identification power of our model on both the latent space and the folding space. Finally, we generalized and validated our approach to other clinical brain disorders in other regions. Our results open up several avenues of work such as the definition of new synthetic benchmarks that match the characteristics of other known anomalies, the use of other deep learning models that exploit the presence of outliers in the training set, or the use of our framework to better understand the folding process.

## Supplementary Material

Supplementary Material

## Data Availability

The Human Connectome Project dataset is a public dataset (Van Essen et al., 2013). The dataset used to generate the masks is available at https://brainvisa.info/data/sulci_database/base_62/2019/. The CCD subjects and the patients suffering from FCD2 are not available for disclosure due to medical confidentiality. The code is available at https://github.com/neurospin-projects/2022_lguillon_rare_folding_detection.
