# Supplementary material for: Identification of rare cortical folding patterns using unsupervised deep learning
Source: Imaging Neurosci (Camb). 2024 Feb 6;2:imag-2-00084. doi: 10.1162/imag_a_00084 (PMC12224473; doi:10.1162/imag_a_00084)
Supplement: Supplementary Material [file imag_a_00084-supp.pdf]

Supplementary Information

Annex 1. Distance maps normalization

We normalize the distance maps to have values between [0, 1], with the highest values on the folds and a saturation at about 4-5 mm. As a matter of fact, we observe that skeleton voxels equal 0 in the initial distance maps X and the values increase with the distance to a sulcus, possibly ranging up to 10 mm. Potential reconstruction errors near the sulci would be minor compared to the voxels located far from them at the edge of the mask, whereas we wish the model to concentrate more on the sulci. Thus, to limit the impact of distance variability far from the sulci, we perform a normalization according to supplementary equation 1, resulting in values between [0, 1], with the highest values on the folds and a saturation at about 4-5 mm which corresponds to half of the typical width of gyri. An example is shown in the supplementary figure 1.

Supplementary equation 1: $$X_{norm} = 1 - [2\frac{1}{1+e^{-X}} - 1]$$

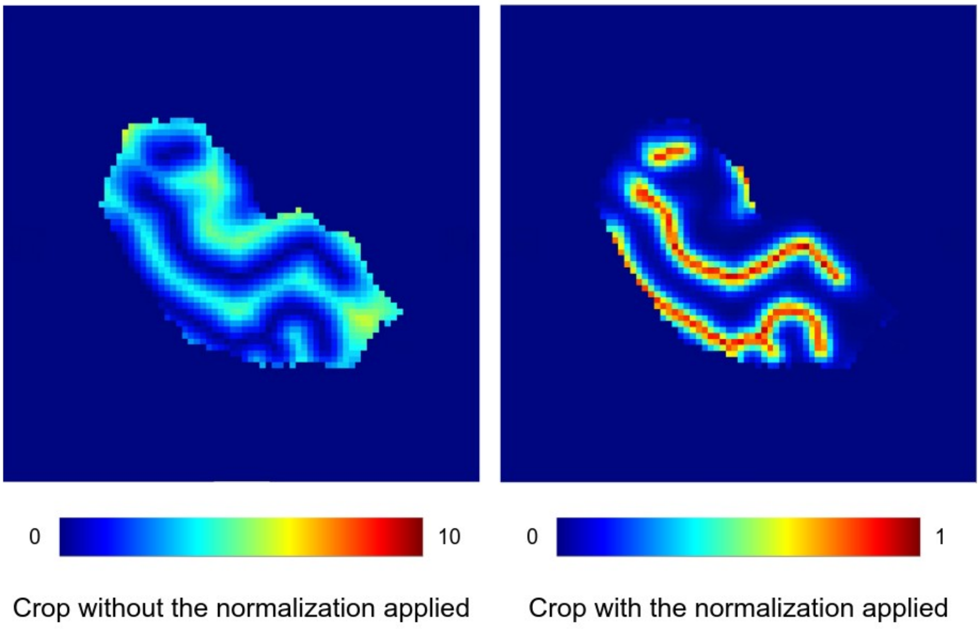

Supplementary figure 1: Crops without (left) and with (right) the normalization applied

Example of an input crop without and with the normalization applied and presented in section 2.3.2.

Annex 2: Description of skeleton's folds nature

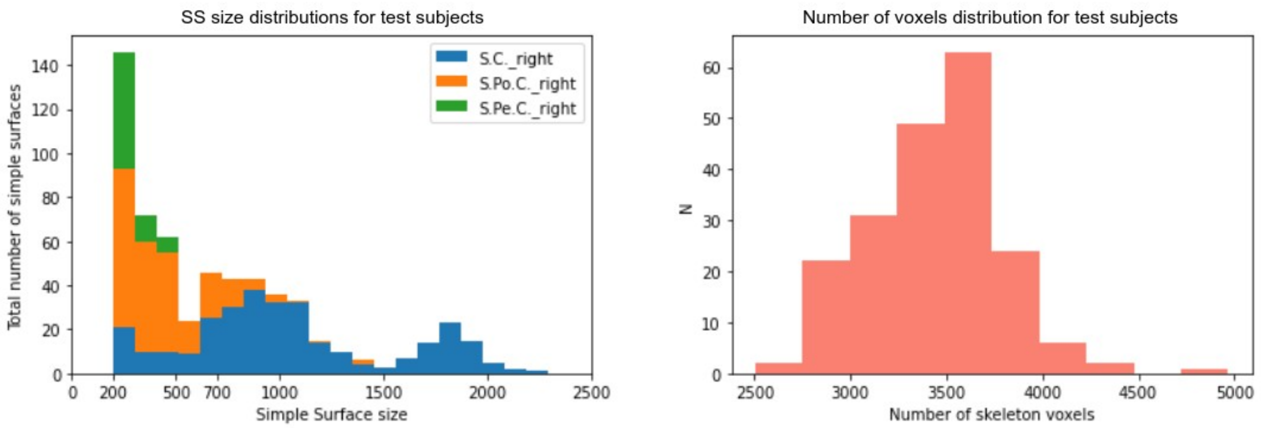

Supplementary figure 2: Description of the skeletons of the test set

Left: Stacked histogram representing the distribution of simple surfaces sizes for the test subjects for the three main sulci of our crop, the central sulcus (S.C._right), the precentral sulcus (S.Pe.C._right) and the postcentral sulcus (S.Po.C._right). (Note: The labeling used is automatic and therefore not entirely reliable, but these labels are sufficient to draw conclusions regarding the SS size distribution.)

Right: Distribution of the number of skeletons' fold voxels for the test subjects in our crops.

Annex 3: Distance maps and folds visualization

Data visualization, and shape characterization in particular, can be performed directly based on the distance maps. However, this type of input enables only 2D slice views. To better visualize the folds of our crops, we binarize our distance maps with an empirically defined threshold of 0.4 and convert them to meshes.

Annex 4: beta-VAE hyperparameter selection

We seek to choose the best values for the Kullback-Liebler divergence weight (beta) and the number of dimensions of the latent space (L) in the task of identifying interrupted central sulci.

To select the best values we used two criteria, the reconstruction quality and the detection power on a proxy for interrupted central sulci. The precentral and postcentral sulci have some similarities with the central sulcus regarding orientation, size and shape. In addition, they are usually more interrupted and have more ramifications which make them a good proxy in the task of interrupted central sulci detection.

However, due to the interruptions and the ramifications, the classification between a central sulcus and a pre or postcentral sulcus may be very easy for some subjects. In order to make the classification task non trivial and thus informative, we selected the subjects for whom the task is harder, i.e. that have a pre or a postcentral sulcus that looks like the central sulcus.

Therefore, our hyperparameter selection method is based on three steps:

- generating the pre and post central sulci crops
- identifying the precentral and postcentral sulci that present some ambiguities with the central sulcus,
- the actual gridsearch.

<u>These three steps are detailed in the following</u>:

1) Generation of the pre and post central sulci crops

To obtain crops of these two other sulci, like presented in the section 2.2. for the central sulcus, we generated distance maps of the whole hemisphere based on the folding graph of the HCP subjects. Then, like for the central sulcus, we learned masks of the precentral and of the postcentral sulci. In order to have the same image dimensions as the ones for the central sulcus, we adapted the mask to the adequate dimensions by adding or deleting a few voxels. Therefore, we obtain 3D crops of the precentral sulcus and of the postcentral sulcus.

2) "Ambiguous" precentral and postcentral sulci identification

The aim of this second step is to identify "ambiguous" precentral and postcentral sulci. In order to do this, we trained a beta-VAE (L = 150 and beta = 1) on the train set described in 2.3.2 (only control central sulcus crops). Once the model was trained, we encoded all the training central sulci and all the precentral and postcentral sulci. We then trained two linear SVM to classify the latent codes between the central and precentral sulci, and between central and postcentral sulci. All precentral and postcentral sulci that were wrongly predicted as central sulci were considered as ambiguous. We repeated these steps five times in order to increase the robustness. We found 28 ambiguous precentral sulci and 18 ambiguous prostcentral sulci.

3) Gridsearch

Once we identified ambiguous subjects, we consider them as a proxy for interrupted central sulci, i.e. as the rare patterns to detect. We then performed the gridsearch to find the best hyperparameter configuration to detect them. We trained a beta-VAE on the train set described in 2.3.2 (only control central sulcus crops) for each configuration. Next we encoded the central sulci of the validation set

and the ambiguous pre and postcentral sulci. We trained a linear SVM to classify between the latent codes of the validation samples and of the outliers which are composed of the pre and post central sulci.

Annex 5: Generation of the deletion benchmarks

Deletion benchmark consists of subjects for whom we have erased one simple surface (SS). Regarding the generation process, deleting simple surfaces directly on the distance maps would not be interesting as the voxels next to the simple surface indicate the SS position. To tackle this issue, the suppression was done during the generation of the raw skeletons. The distance map was then computed based on the pruned skeletons, according to the methodology presented in the paragraph "*From skeletons to distance maps*".

Annex 6: Asymmetry benchmarks

To further highlight the main differences between the two hemispheres, we selected the most important dimensions for the classifier, here dimensions 9 and 36. In Fig.3A., control and benchmark data are represented according to these two dimensions. Even if the separation is not well marked, we can observe a trend represented by the arrow. To better understand the asymmetry characteristics encoded by the model, we analysed the features encoded by the 9th dimension. To do so, we took the average for all 75 dimensions of the latent space, and we travelled from the minimum to the maximum of the 9th dimension and reconstructed the resulting vector. Fig. 3B. 1, 2, and 3 represent the reconstructions. These interpolations confirm the trend observed previously. We observe a double-knob configuration in the left hemisphere. The view from underneath and the side view enable visualizing the pli de passage frontal moyen (PPFM). A pli de passage is a gyrus that connects two gyri and which is buried in the depth of some furrows (Mangin et al., 2019). Fig.2B.1. and 2. in the main article propose a visualization of a ”pli de passage” located in the central sulcus, the PPFM. According to the different views from Fig.3B. 1, 2 and 3, it seems that the PPFM is smaller in the right hemisphere and located higher in the central sulcus.

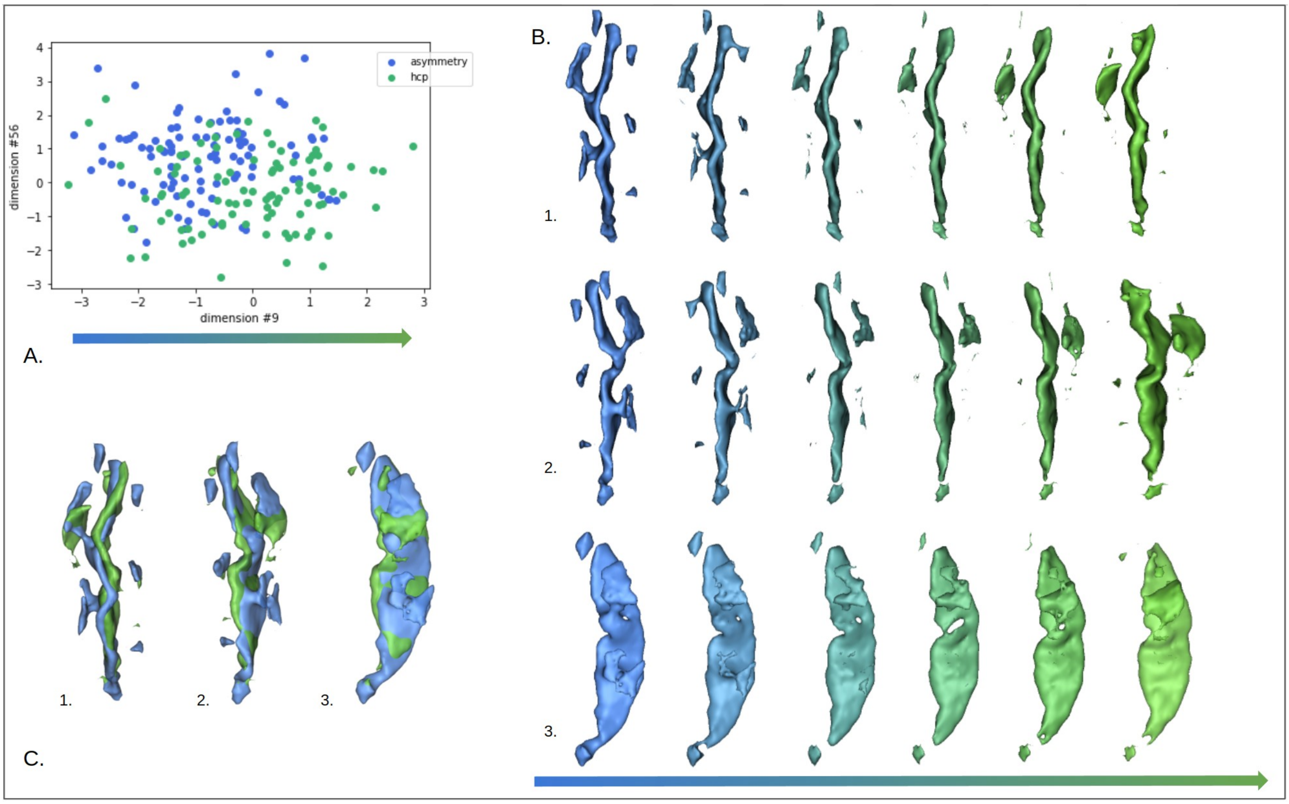

Supplementary figure 3: Travelling through the 9th dimension of the latent space. A. Visualization of controls and asymmetry benchmark according to the most important features of the classifier. B. Interpolations along the 9th dimension. 1, 2, and 3, respectively correspond to the upper, bottom and side view of these interpolations. C. Superposition of extreme interpolations.

Having the PPFM successfully encoded constitutes another element suggesting the relevance of the learned representations. Indeed, this pli de passage was first described in 1888 (Broca et al., 1888) and has been a source of growing interest due to its link with the motor hand area (Boling et al., 2004) and in the context of understanding the formation of the knob regarding evolutionary questions (Hopkins et al., 2014). Our model was able to encode the PPFM in the latent space as well as its asymmetry characteristics. Indeed, we observed that the PPFM is smaller in the right hemisphere which corresponds to central sulcus depth variations described in (Amunts et al., 1996). This is also consistent as the PPFM has been correlated to the hand. Therefore, right-handed subjects tend to have a more developed hand area in the left hemisphere and thus a larger PPFM.

Annex 7: Analysis of control subjects considered as outliers

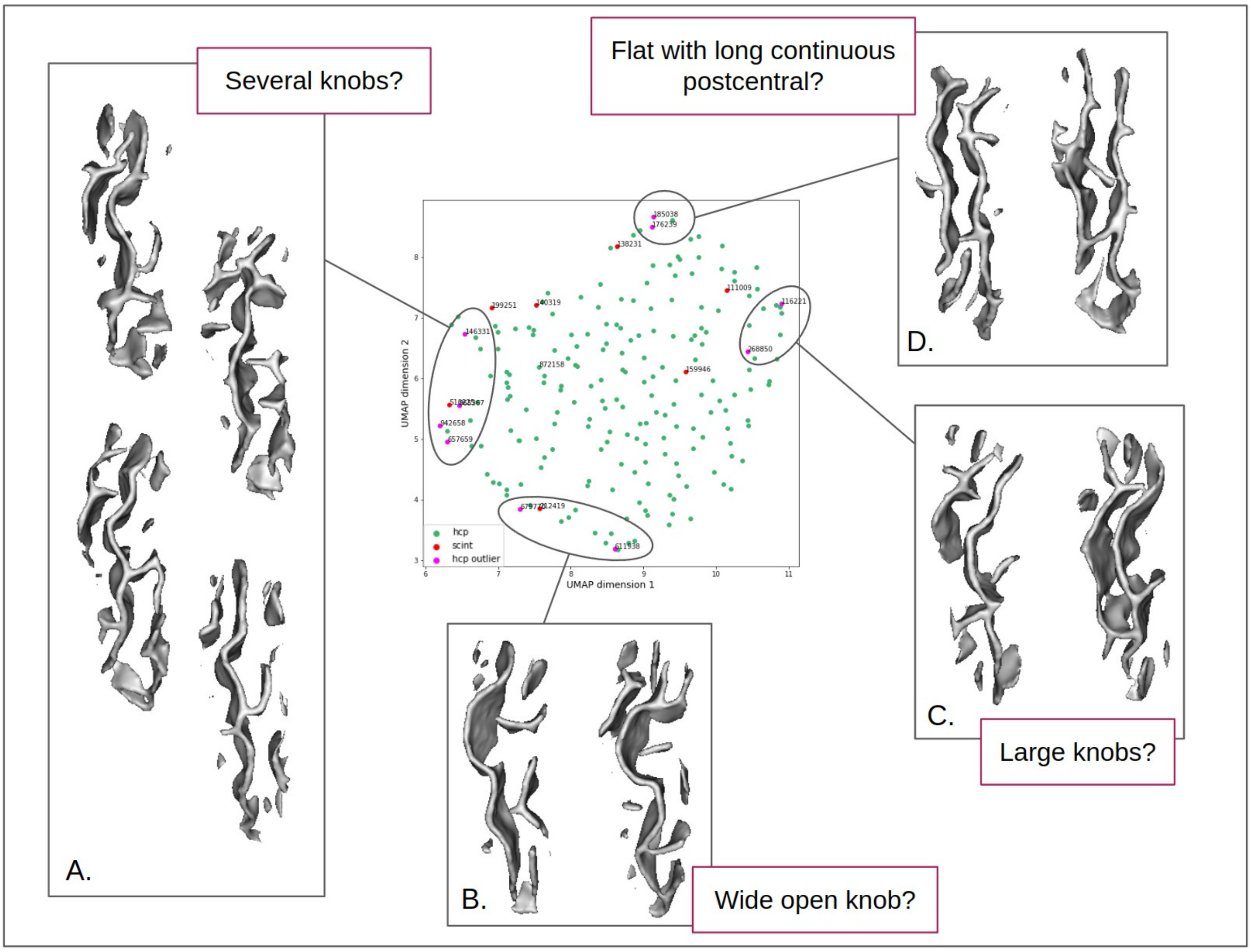

Supplementary figure 4: Control subjects identified as outliers. A, B, C and D correspond to groups of visually similar patterns. The UMAP projection is the same as the one in Fig.9. Control subjects identified as outliers are in pink and subjects with interrupted central sulci are still represented in red.

Based on the results of the interrupted central sulcus in the UMAP space, we have seen that some other patterns in the control population were at the margin of the representation and could be considered as outliers. Interrupted central sulci may not be the rarest pattern, and other folding configurations may be very scarce. Therefore, we also looked at control subjects repeatedly predicted as outliers by these algorithms.

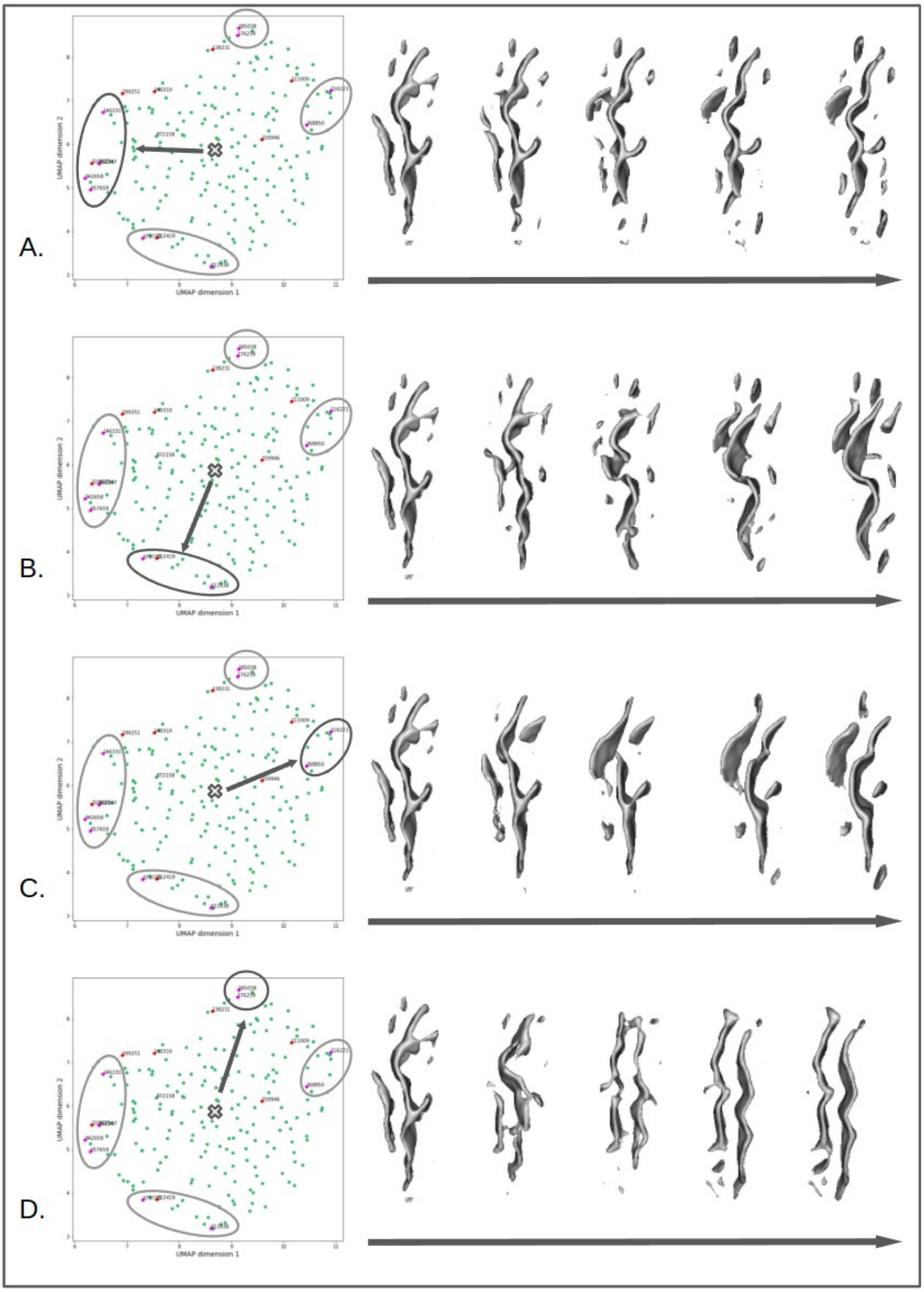

Supplementary figure 5: Travelling through the latent space from the centroid to the margin of the UMAP space. The centroid is the centroid of HCP controls. Then, for each row, interpolations between the centroid and one of the patterns of each group are computed and then reconstructed.

Fig.4 presents the controls' patterns most often predicted as outliers by the OCSVM. First, we note that the outliers are logically located at the border of the distribution. Moreover, we observe distinct patterns in different regions of the UMAP space. We visually highlighted the subjects of the four

regions. Analysing the corresponding crops' meshes, we observe similarities within the groups. Group B seems to demonstrate a very wide open knob. In addition, the knobs are well defined by the upper and the bottom part of the sulcus. On the contrary, the sulci of group C appear to have larger knobs than usual but they show more continuity with the upper and the bottom parts. The pattern of group D seems to correspond to a rather flat central sulcus with a close, long and continuous postcentral sulcus. The shape characteristics of A are less obvious but the sulci give the impression of having several small knobs, two or even three in the two bottom cases and a small part of the precentral inferior opposite to an upper part of the postcentral sulci.

Fig.5 provides a better understanding of these features. For each pattern, we go from the centroid to one of the subjects in each group by interpolating and generating samples. Fig.5A. presents the interpolations from the centroid to the several-knobs pattern. We gradually see the upper part of the hand knob curving and becoming more pronounced until forming a first knob at the top of the sulcus. Another knob in the bottom part appears similarly. Likewise, patterns B, C and D vary progressively until they match the centroid's shape.

These results show that our model was able to propose four other groups of likely rare patterns. The pattern representing a rather flat central sulcus is indeed a non-typical configuration. Less than 2% of the studied subjects were reported to have such a configuration in (Caulo et al., 2007). Moreover, flat central sulci appeared as the most important feature when comparing controls to congenital one-handed subjects who tended to demonstrate flatter central sulci (Sun et al., 2017), confirming that flat central sulci are less frequent patterns. The groups representing large knobs and wide open knobs (Fig.4B. and C.) are also an atypical configuration that is present at one extremity of the axis representing the most extreme variations in Human and is closer to configurations we observe in Chimpanzees (Foubet et al., 2022).

Nevertheless, the identification of these control subjects as outliers highlights the complexity of defining "being-rare". In this case, one solution might be to adopt a data-driven way, in which, for example, these four categories of outlier control subjects could be added to the target cases and analyses carried out iteratively.

Annex 8: Reconstructions and residuals for the patients suffering from FCD2 and controls

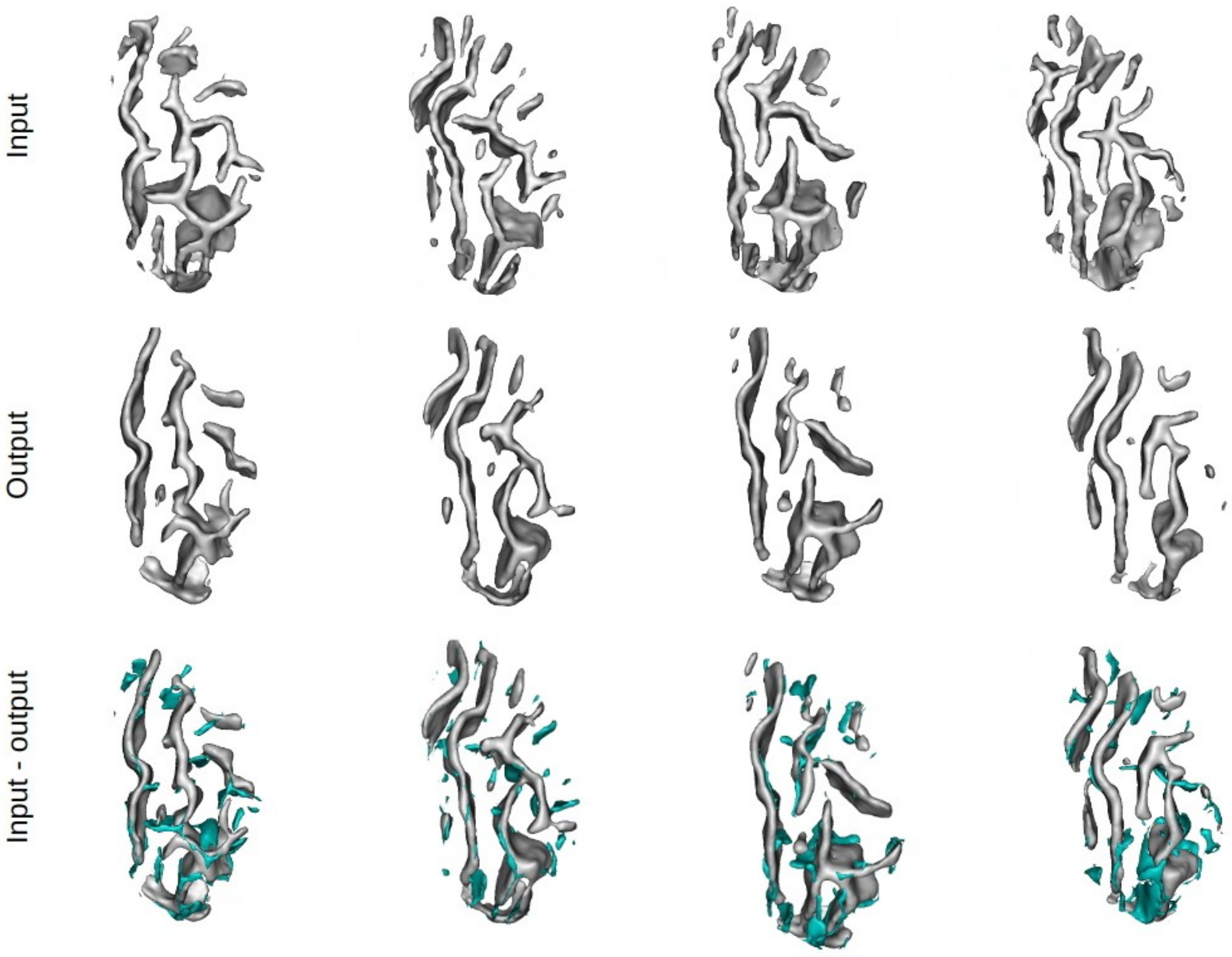

(a) Control subjects

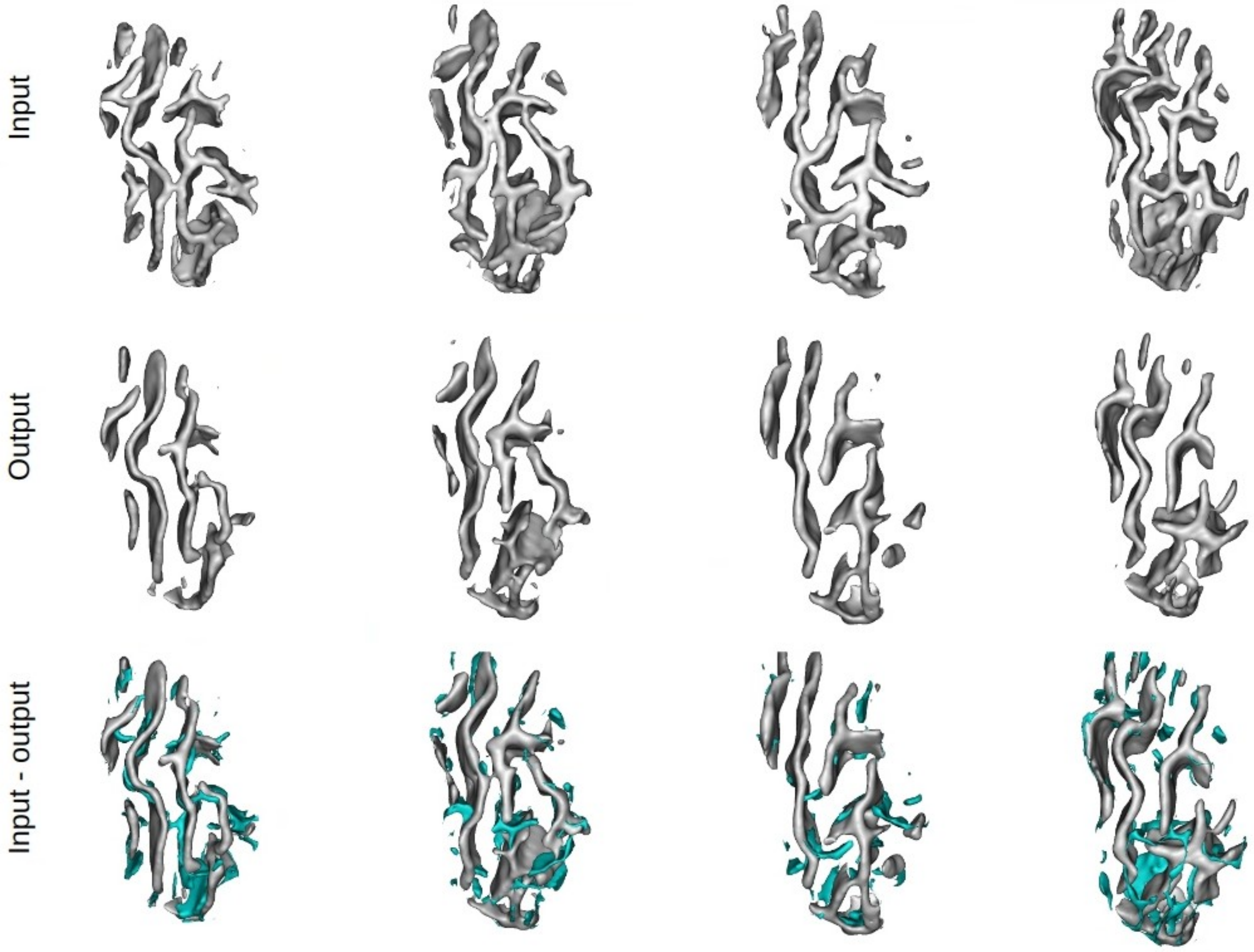

(b) Patients with a positive MRI

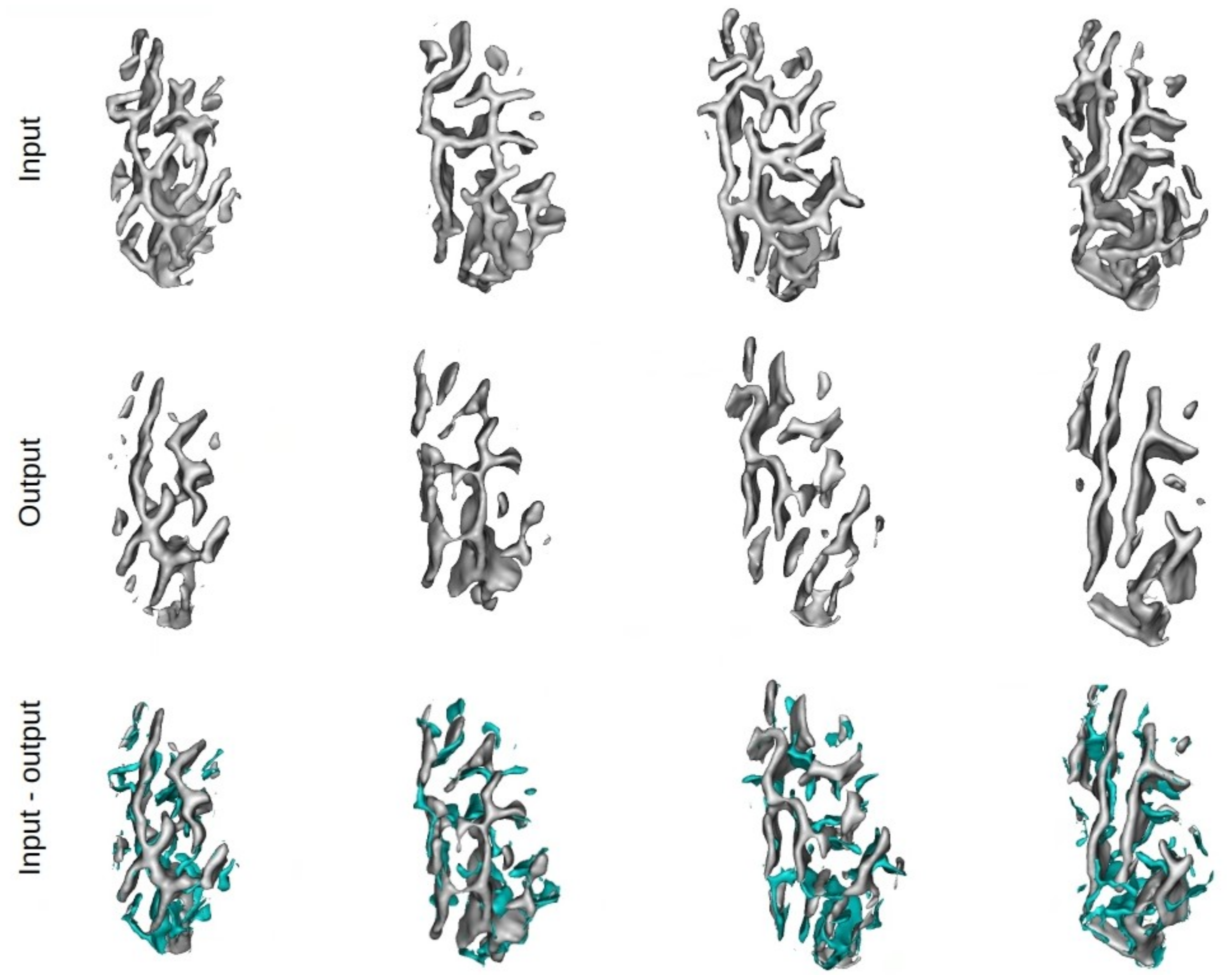

(c) Patients with a negative MRI

Supplementary figure 6: Right central region reconstructions and residuals for the patients suffering from FCD2 and controls. Each column corresponds to a subject. For all rows, distance maps are converted to meshes for easier visualization. First row: input data. Second row: reconstruction of the model. Third row: Reconstructions of the model with the difference between the input and the output, i.e. the model's omissions. Note that all patients represented have the lesion located in the right hemisphere.

Annex 9: Comparison between skeleton-based images and distance maps based images

In this work, we studied cortical folding patterns based on two types of input. First, we used binary skeleton images (Fig. 7A.) to identify typical patterns that we then converted to distance maps (Fig. 7B.) to characterize rare folding patterns. We proposed distance maps in order to limit some shortcomings of the skeletons. To assess whether the distance maps are indeed more suited we

compared the results obtained using skeletons and distance maps. We analyzed both the latent and the folding space on our deletion and asymmetry benchmarks. Note that the deletion benchmarks are slightly different from those presented in the article as the method to generate them was later improved (the latest version is used in the article).

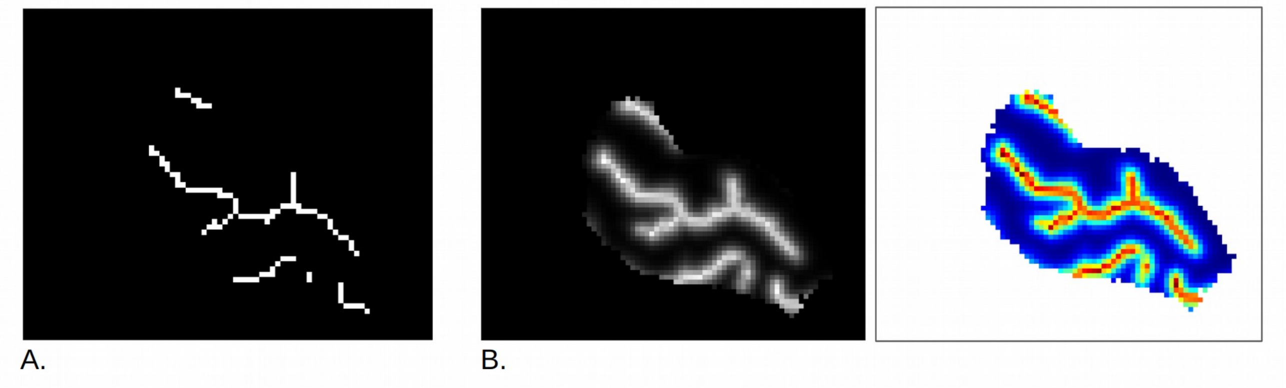

Supplementary figure 7: Skeletons and distance maps

A: Skeleton. B. Distance maps in levels of grey or in colours for easier visualization. Note that only a slice is represented for visualization but both the inputs are 3D volumes.

In the latent space, the fake anomalies seem to be distributed more differently when using distance maps than skeletons for deletion 700 and 1000 (Fig. 8). For smaller deleted SS, it seems that there is no difference. Regarding the ROC curves, they are slightly better with skeletons for small erased SS (deletion 200 and 500) but the results are equivalent for deletion 700 and a bit better with distance maps for deletion 1000. In the case of benchmark 200, we are not particularly interested in having the small deleted SS identified because they mainly concern the inter-subject variability of small branches instead of actual rare configurations. In addition, these SS generally correspond to the precentral or postcentral sulci, which are not our object of interest. This could be due to the fact that in distance maps, unlike skeletons, some isolated branches located at the edges of the crop may be more related to the rest of the crop due to the information on the distance to the nearest sulcus held by all voxels, particularly thanks to the applied rotations. Note that for benchmark 500, like in the article, we have only 34 subjects in each class which limits reliable conclusions.

In return, in the folding space, we observe greater differences between the distributions of the reconstruction errors of controls and benchmarks using distance maps than skeletons (Fig.10).

For benchmark asymmetry, results are roughly similar using both inputs (Fig. 11). However, we observe that the averages generated based on the distance maps (Fig.12) are much more precise. On the contrary, those based on the skeletons are thicker which makes it difficult to analyze properly the differences.

To conclude, distance maps seem to lead to equivalent or better results, particularly in the folding space. Furthermore, to visualize the distance maps in 3D a threshold has to be applied, which allows for better-quality images. Future works could improve the distance maps generation process. Indeed, the current method works but could be improved by generating the distance maps on the fly by convolving a Gaussian directly over the skeletons.

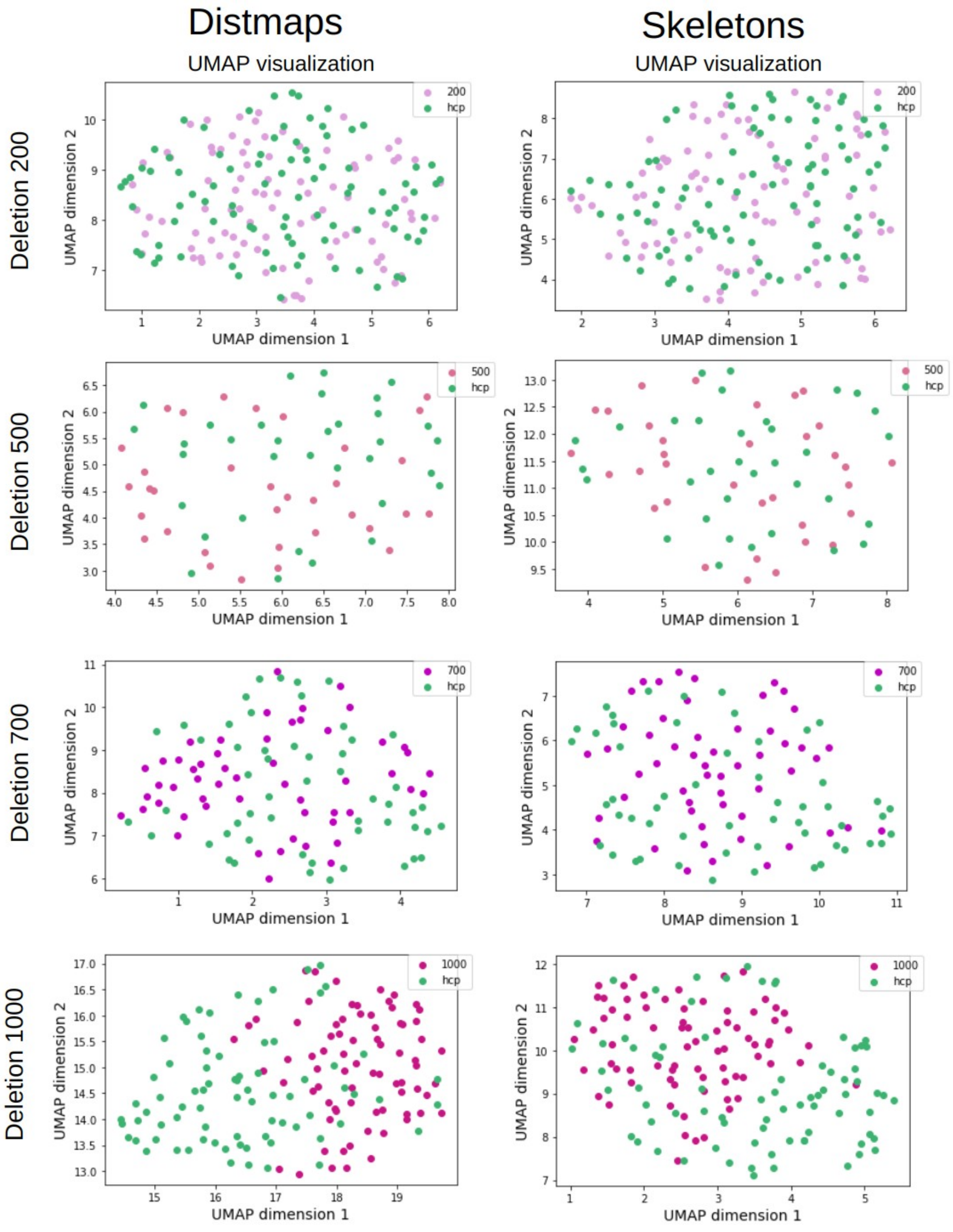

Supplementary figure 8: Comparison of UMAP projections of deletion benchmarks for skeletons and distance maps

For both columns, each row corresponds to one benchmark. Left: UMAP projections obtained with distance maps as input. Right: UMAP projections obtained with skeletons as input.

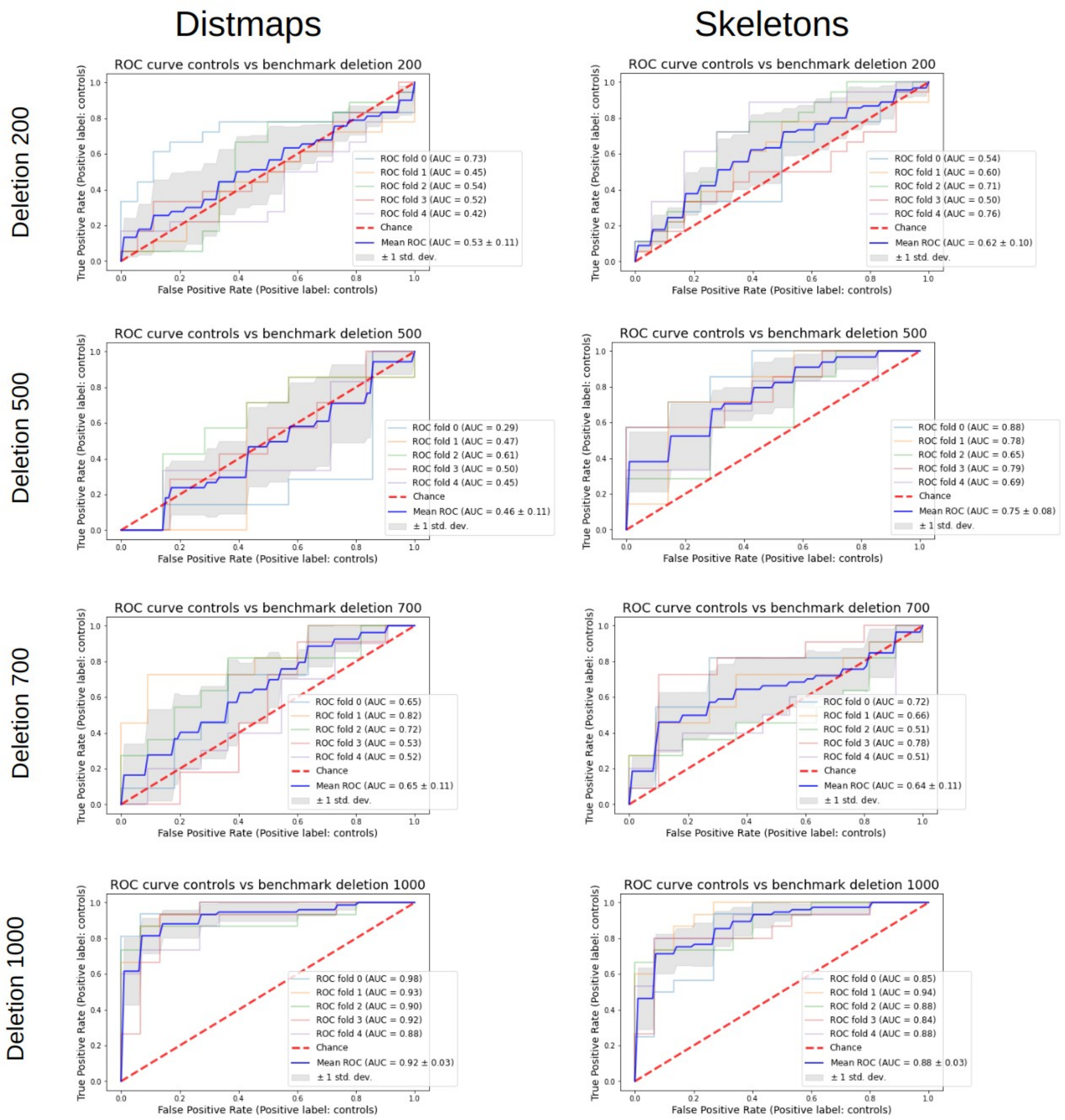

Supplementary figure 9: Comparison of ROC curves of deletion benchmarks for skeletons and distance maps

For both columns, each row corresponds to one benchmark. Left: ROC obtained with distance maps as input. Right: ROC obtained with skeletons as input.

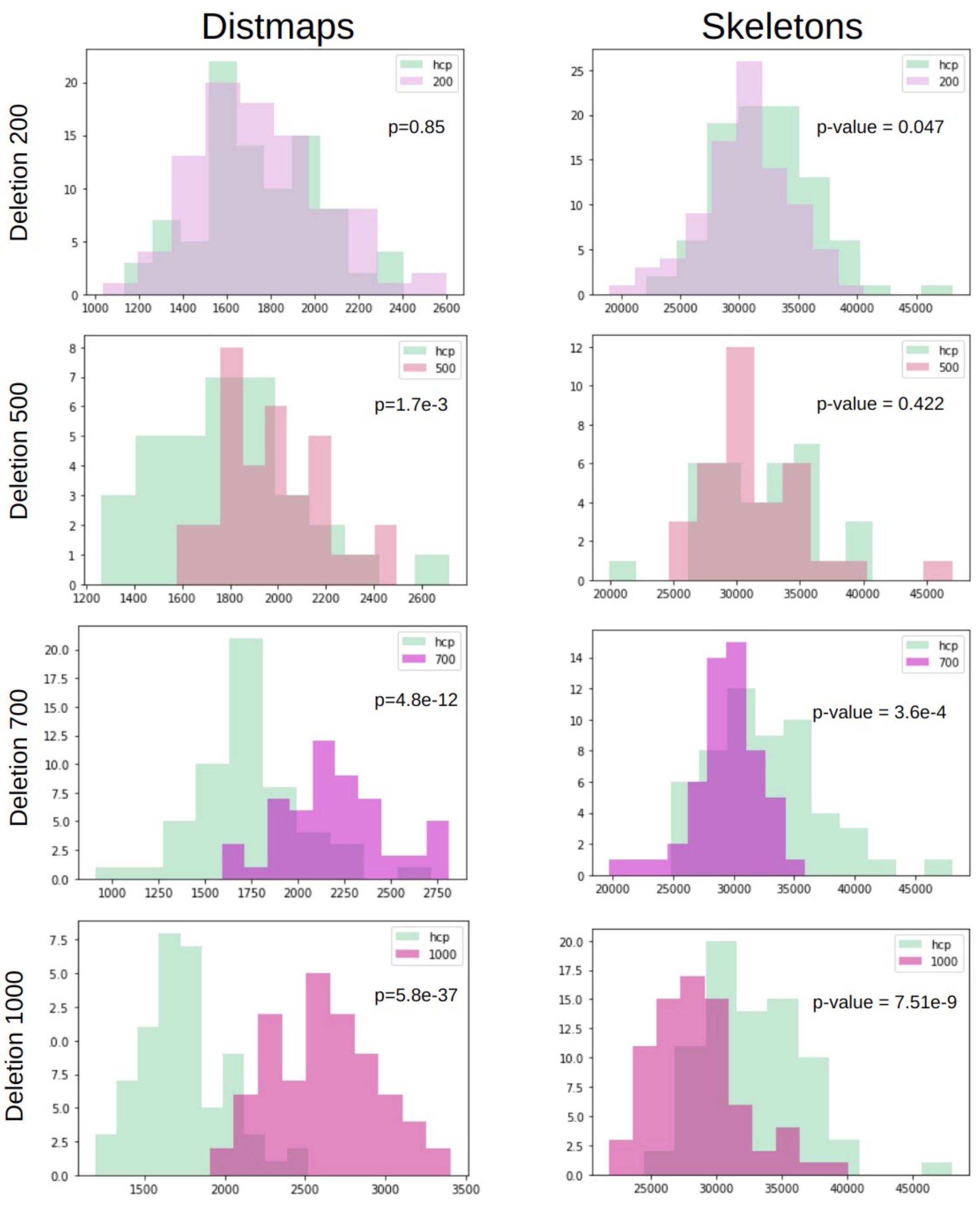

Supplementary figure 10: Comparison of reconstruction error distributions of deletion benchmarks for skeletons and distance maps

For both columns, each row corresponds to one benchmark. Left: Reconstruction error distributions obtained with distance maps as input. Right: Reconstruction error distributions obtained with skeletons as input.

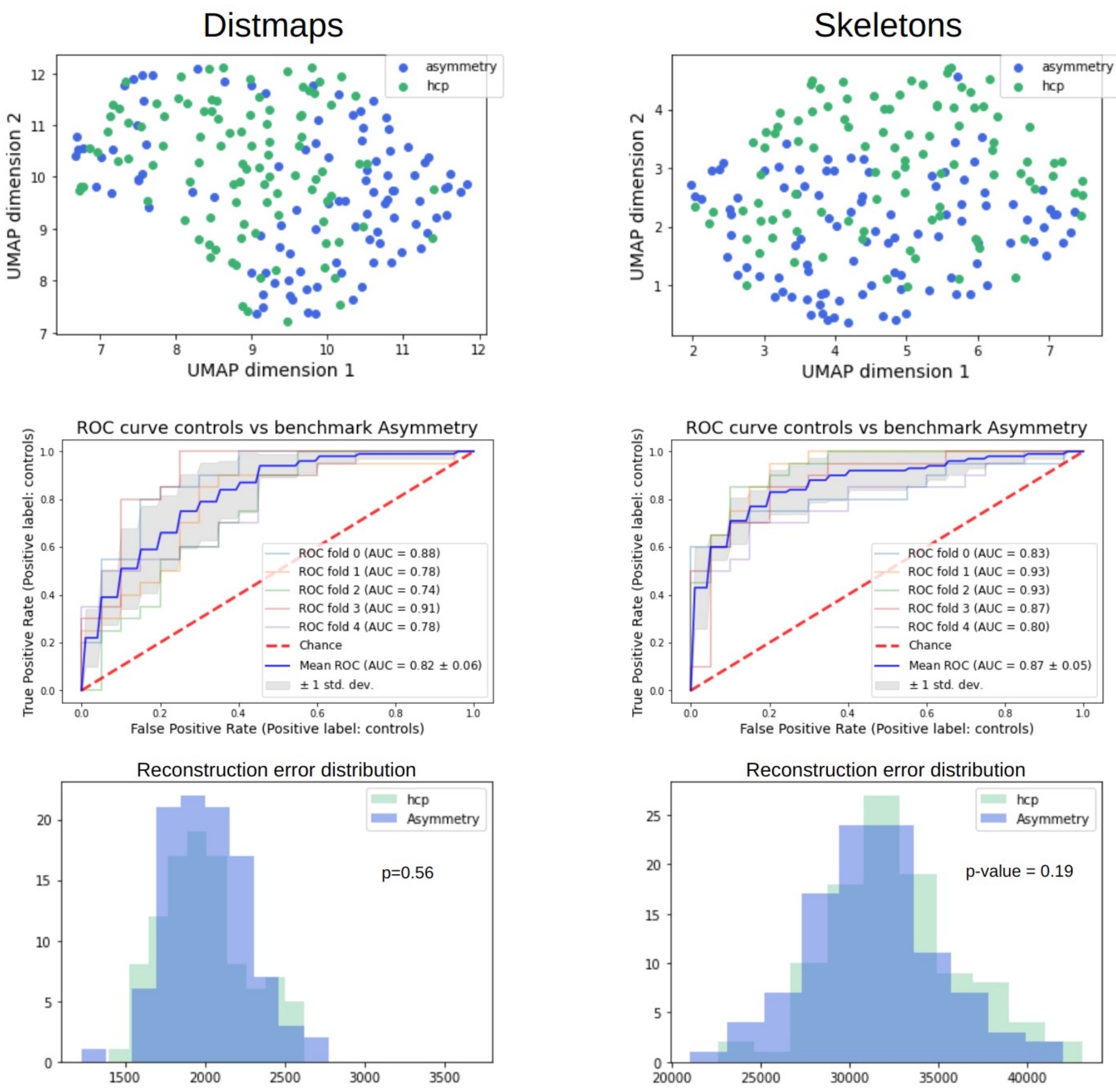

Supplementary figure 11: Comparison of asymmetry benchmark results for distance maps and skeletons

UMAP projection of benchmark and control data, ROC curves of classification of control and benchmark data, and reconstruction error distributions

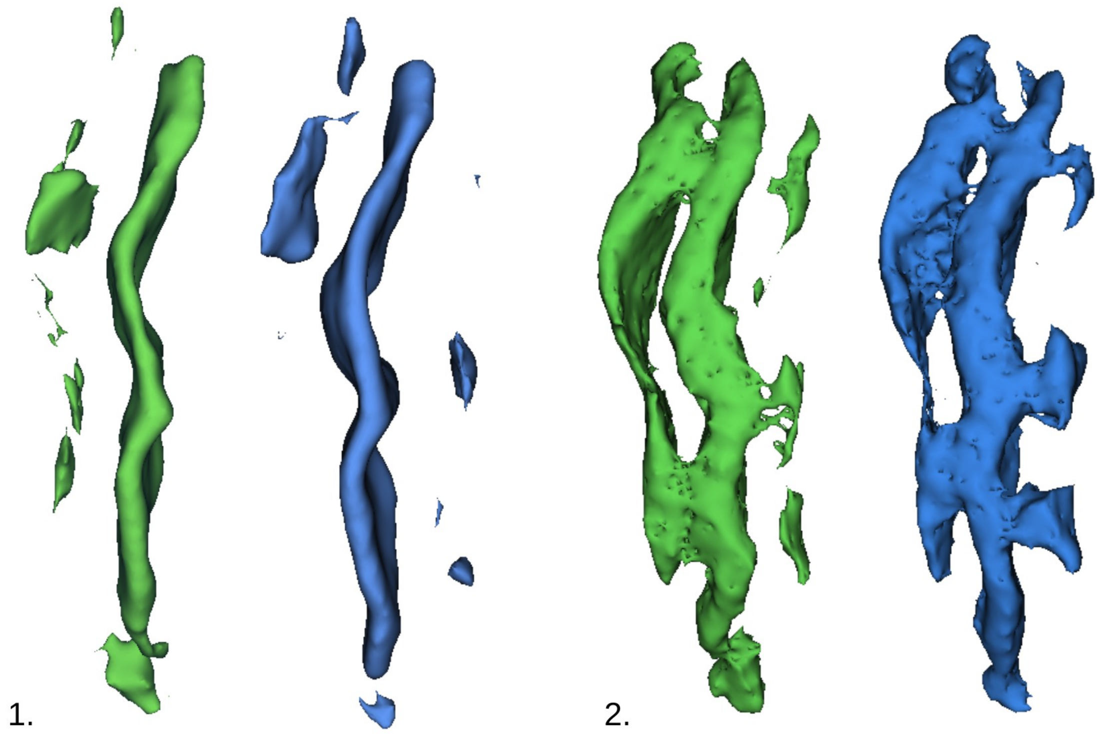

Supplementary figure 12: Comparison of asymmetry benchmark average patterns for distance maps and skeletons

Averages for control subjects i.e. right hemispheres (in green), and for the asymmetry subjects, i.e. left hemispheres (in blue). 1. Averages obtained using distance maps as inputs and 2. using skeletons as inputs.

Bibliography

Amunts, K., Schlaug, G., Schleicher, A., Steinmetz, H., Dabringhaus, A., Roland, P.E., Zilles, K., 1996. Asymmetry in the Human Motor Cortex and Handedness. NeuroImage 4, 216–222. https://doi.org/10.1006/nimg.1996.0073

Boling, W.W., Olivier, A., 2004. Localization of hand sensory function to the pli de passage moyen of Broca. J Neurosurg 101, 278–283. https://doi.org/10.3171/jns.2004.101.2.0278

Broca, P., Pozzi, S.J., 1888. Mémoires sur le cerveau de l'homme et des primates. C. Reinwald.

Caulo, M., Briganti, C., Mattei, P.A., Perfetti, B., Ferretti, A., Romani, G.L., Tartaro, A., Colosimo, C., 2007. New morphologic variants of the hand motor cortex as seen with MR imaging in a large study population. AJNR Am J Neuroradiol 28, 1480–1485. https://doi.org/10.3174/ajnr.A0597

Foubet, O., Sun, Z.Y., Hopkins, W., Mangin, J.-F., 2022. Comparison of the shape of the Central Sulcus in Hominids, in: Organisation for Human Brain Mapping. Presented at the Organisation for Human Brain Mapping, Glasgow, United Kingdom.

Hopkins, W.D., Meguerditchian, A., Coulon, O., Bogart, S., Mangin, J.-F., Sherwood, C.C., Grabowski, M.W., Bennett, A.J., Pierre, P.J., Fears, S., Woods, R., Hof, P.R., Vauclair, J., 2014. Evolution of the central sulcus morphology in primates. Brain Behav Evol 84, 19–30. https://doi.org/10.1159/000362431

Mangin, J.-F., Le Guen, Y., Labra, N., Grigis, A., Frouin, V., Guevara, M., Fischer, C., Rivière, D., Hopkins, W.D., Régis, J., Sun, Z.Y., 2019. “Plis de passage” Deserve a Role in Models of the Cortical Folding Process. Brain Topogr 32, 1035–1048. https://doi.org/10.1007/s10548-019-00734-8

Sun, Z.Y., Cachia, A., Rivière, D., Fischer, C., Makin, T., Mangin, J.-F., 2017. Congenital unilateral upper limb absence flattens the contralateral hand knob, in: Organisation for Human Brain Mapping. Presented at the Organisation for Human Brain Mapping, Vancouver, Canada.
